# Baseline Features and Reasons for Nonparticipation in the Colonoscopy Versus Fecal Immunochemical Test in Reducing Mortality From Colorectal Cancer (CONFIRM) Study, a Colorectal Cancer Screening Trial

**DOI:** 10.1001/jamanetworkopen.2023.21730

**Published:** 2023-07-11

**Authors:** Douglas J. Robertson, Jason A. Dominitz, Alexander Beed, Kathy D. Boardman, Barbara J. Del Curto, Peter D. Guarino, Thomas F. Imperiale, Andrew LaCasse, Meaghan F. Larson, Samir Gupta, David Lieberman, Beata Planeta, Aasma Shaukat, Shanaz Sultan, Stacy B. Menees, Sameer D. Saini, Philip Schoenfeld, Stephan Goebel, Erik C. von Rosenvinge, Gyorgy Baffy, Ildiko Halasz, Marcos C. Pedrosa, Lyn Sue Kahng, Riaz Cassim, Katarina B. Greer, Margaret F. Kinnard, Divya B. Bhatt, Kerry B. Dunbar, William V. Harford, John A. Mengshol, Jed E. Olson, Swati G. Patel, Fadi Antaki, Deborah A. Fisher, Brian A. Sullivan, Christopher Lenza, Devang N. Prajapati, Helen Wong, Rebecca Beyth, John G. Lieb, Joseph Manlolo, Fernando V. Ona, Rhonda A. Cole, Natalia Khalaf, Charles J. Kahi, Divyanshoo Rai Kohli, Tarun Rai, Prateek Sharma, Jiannis Anastasiou, Curt Hagedorn, Ronald S. Fernando, Christian S. Jackson, M. Mazen Jamal, Robert H. Lee, Farrukh Merchant, Folasade P. May, Joseph R. Pisegna, Endashaw Omer, Dipendra Parajuli, Adnan Said, Toan D. Nguyen, Claudio Ruben Tombazzi, Paul A. Feldman, Leslie Jacob, Rachel N. Koppelman, Kyle P. Lehenbauer, Deepak S. Desai, Mohammad F. Madhoun, William M. Tierney, Minh Q. Ho, Heather J. Hockman, Christopher Lopez, Emily Carter Paulson, Martin Tobi, Hugo L. Pinillos, Michele Young, Nancy C. Ho, Ranjan Mascarenhas, Kirrichai Promrat, Pritesh R. Mutha, William M. Pandak, Tilak Shah, Mitchell Schubert, Frank S. Pancotto, Andrew J. Gawron, Amelia E. Underwood, Samuel B. Ho, Priscilla Magno-Pagatzaurtundua, Doris H. Toro, Charles H. Beymer, Andrew M. Kaz, Jill Elwing, Jeffrey A. Gill, Susan F. Goldsmith, Michael D. Yao, Petr Protiva, Heiko Pohl, Tassos Kyriakides

**Affiliations:** 1 VA Medical Center, White River Junction, Vermont; 2 Geisel School of Medicine at Dartmouth, Hanover, New Hampshire; 3 VA Puget Sound Health Care System, Seattle, Washington; 4 University of Washington School of Medicine, Seattle; 5 Cooperative Studies Program Coordinating Center, VA Connecticut Healthcare System, West Haven, Connecticut; 6 Department of Veterans Affairs Cooperative Studies Program Clinical Research Pharmacy Coordinating Center, Albuquerque, New Mexico; 7 Statistical Center of HIV/AIDS Research and Prevention, Fred Hutchinson Cancer Center, Seattle, Washington; 8 Center for Innovation, Health Services Research and Development, Richard L. Roudebush VA Medical Center and Department of Medicine, Indiana University School of Medicine, Indianapolis; 9 Section of Gastroenterology, VA San Diego, and Department of Medicine, University of California, San Diego; 10 Division of Gastroenterology and Hepatology, Portland VA Medical Center, and Oregon Health and Science University, Portland; 11 New York Harbor VA Healthcare System and New York University Grossman School of Medicine, New York; 12 Division of Gastroenterology, Hepatology, and Nutrition, University of Minnesota, Minneapolis VA Healthcare System, Minneapolis; 13 Division of Gastroenterology, Department of Internal Medicine, Ann Arbor VA Medical Center, Ann Arbor, Michigan; 14 Division of Gastroenterology, Michigan Medicine, Ann Arbor; 15 US Department of Veteran Affairs Health Services Research and Development Center for Clinical Management Research, Ann Arbor, Michigan; 16 Division of Gastroenterology, University of Michigan, Ann Arbor; 17 Institute for Healthcare Policy and Innovation, University of Michigan, Ann Arbor; 18 John D. Dingell VA Medical Center, Atlanta, Georgia; 19 Atlanta VA Medical Center, Decatur, Georgia; 20 Emory University, Atlanta, Georgia; 21 VA Maryland Health Care System, Baltimore; 22 University of Maryland School of Medicine, Baltimore; 23 Department of Medicine, VA Boston Healthcare System, Harvard Medical School, Boston, Massachusetts; 24 Primary Care, West Roxbury, Massachusetts; 25 Boston University, Boston, Massachusetts; 26 Gastroenterology Section, Jesse Brown VA Medical Center, and University of Illinois at Chicago; 27 Louis A. Johnson VA Medical Center, Clarksburg, West Virginia; 28 Department of Surgery, West Virginia University, Morgantown; 29 Department of Medicine, Case Western Reserve University School of Medicine, Cleveland, Ohio; 30 Louis Stokes VA Medical Center, Cleveland, Ohio; 31 VA Northeast Ohio Healthcare System, Cleveland; 32 VA North Texas Health Care Center, University of Texas Southwestern Medical School, Dallas; 33 VA North Texas Healthcare System, University of Texas Southwestern, Dallas; 34 Division of Gastroenterology and Hepatology University of Colorado School of Medicine, Denver; 35 Rocky Mountain Regional VA Medical Center, University of Colorado Anschutz Medical Center, Aurora; 36 Division of Gastroenterology and Hepatology, Rocky Mountain Regional VA Medical Center, University of Colorado Anschutz Medical Center, Aurora; 37 John D. Dingell VA Medical Center and Wayne State University School of Medicine, Detroit, Michigan; 38 Durham VA Medical Center, Durham, North Carolina; 39 Division of Gastroenterology, Durham VA Medical Center, Durham, North Carolina; 40 Division of Gastroenterology, Duke University Medical Center, Durham, North Carolina; 41 New Jersey VA Healthcare System, East Orange; 42 VA Central California Health Care System, University of California, San Francisco, Fresn; 43 Department of Medicine, University of Florida, Gainesville; 44 Division of Gastroenterology, University of Florida, Gainesville; 45 Malcolm Randall VA Medical Center, Gainesville, Florida; 46 VA Pacific Islands Health Care System, Honolulu, Hawaii; 47 University of Hawaii School of Medicine, Honolulu; 48 Department of Gastroenterology, Michael E. DeBakey VA Medical Center, Houston, Texas; 49 Center for Innovations in Quality, Effectiveness, and Safety, Michael E. DeBakey VA Medical Center, Houston, Texas; 50 Section of Gastroenterology and Hepatology, Department of Medicine, Baylor College of Medicine, Houston, Texas; 51 Richard L. Roudebush VA Medical Center, Indiana University School of Medicine, Indianapolis; 52 Kansas City VA Medical Center, Kansas City, Missouri; 53 Providence Sacred Heart Medical Center, Spokane, Washington; 54 Borland Groover Clinic, Jacksonville, Florida; 55 Division of Gastroenterology and Hepatology, University of Kansas School of Medicine, Kansas City; 56 Division of Gastroenterology and Hepatology, Kansas City VA Medical Center, Kansas City, Missouri; 57 Central Arkansas Veterans Healthcare System, Gastroenterology and Hepatology Division, University of Arkansas for Medical Sciences, Little Rock; 58 Gastroenterology Division, New Mexico Veterans Healthcare System, and Department of Medicine, University of New Mexico School of Medicine, Albuquerque; 59 VA Loma Linda Healthcare System, Loma Linda, California; 60 Department of Medicine, University of California, Riverside; 61 Oceana Gastroenterology Associates, Corona, California; 62 VA Long Beach Health Care System, Long Beach, California; 63 University of California, Irvine; 64 V22 Clinical Resource Hub, Long Beach, California; 65 Greater Los Angeles VA Healthcare System, Los Angeles, California; 66 David Geffen School of Medicine at UCLA, Los Angeles, California; 67 Division of Gastroenterology, Hepatology and Parenteral Nutrition, VA Greater Los Angeles Healthcare System, Los Angeles, California; 68 Departments of Medicine and Human Genetics, David Geffen School of Medicine at UCLA, Los Angeles, California; 69 University of Louisville, Louisville, Kentucky; 70 Robley Rex VA Medical Center, Louisville, Kentucky; 71 Division of Gastroenterology, Hepatology and Nutrition, University of Louisville School of Medicine, Louisville, Kentucky; 72 William S. Middleton VA Medical Center, Madison, Wisconsin; 73 Gastroenterology and Hepatology, University of Wisconsin School of Medicine and Public Health, Madison; 74 Memphis VA Medical Center, Memphis, Tennessee; 75 University of Tennessee Health Science Center, Memphis; 76 University of Tennessee, Memphis; 77 Bruce W. Carter VA Medical Center, Miami, Florida; 78 Minneapolis VA Healthcare System, Minneapolis, Minnesota; 79 Northport VA Medical Center, State University of New York Stony Brook, Northport; 80 Oklahoma City VA Medical Center, Oklahoma City; 81 Section of Digestive Diseases and Nutrition, Department of Medicine, University of Oklahoma, Oklahoma City; 82 Department of Infectious Disease, Orlando VA Healthcare System, University of Central Florida, Orlando; 83 Orlando VA Healthcare System, Orlando, Florida; 84 VA Medical Center, Philadelphia, Pennsylvania; 85 University of Philadelphia, Philadelphia, Pennsylvania; 86 Department of Research and Development, John D. Dingell VA Medical Center, Detroit, Michigan; 87 Phoenix VA Healthcare System, Phoenix, Arizona; 88 University of Arizona College of Medicine, Phoenix; 89 Division of Gastroenterology and Hepatology, Portland VA Medical Center, and Oregon Health and Science University, Portland; 90 Central Texas Veterans Health Care System, Austin Outpatient Clinic, Austin, Texas; 91 Department of Medicine, Dell Medical School, The University of Texas at Austin; 92 Providence VA Medical Center, Providence, Rhode Island; 93 The Warren Alpert Medical School of Brown University, Providence, Rhode Island; 94 McGuire VA Medical Center, Richmond, Virginia; Now with The University of Texas Health Science Center, Houston; 95 Richmond VA Medical Center, Richmond, Virginia; 96 Virginia Commonwealth University, Richmond, Virginia; 97 Digestive Disease and Surgery Institute, Cleveland Clinic Florida, Weston; 98 Central Virginia VA Healthcare System, Richmond; 99 Salisbury VA Medical Center, Salisbury, North Carolina; 100 Wake Forrest University School of Medicine, Winston-Salem, North Carolina; 101 Salt Lake City VA Medical Center, Salt Lake City, Utah; 102 University of Utah, Salt Lake City; 103 VA Medical Center San Diego, San Diego, California; 104 Section of Gastroenterology, VA Caribbean Healthcare System, San Juan, Puerto Rico; 105 VA Puget Sound Healthcare System, Seattle, Washington; 106 University of Washington School of Medicine, Seattle; 107 Gastroenterology Section, VA Puget Sound Healthcare System, Seattle, Washington; 108 St Louis VA Medical Center, St Louis, Missouri; 109 Division of Gastroenterology, Washington University School of Medicine, St Louis, Missouri; 110 James A. Haley VA Hospital, Tampa, Florida; 111 University of South Florida College of Medicine, Tampa; 112 Gastroenterology and Hepatology Section, VA Medical Center, Washington, District of Columbia; 113 George Washington University School of Medicine and Health Sciences, Washington, District of Columbia; 114 VA Connecticut Healthcare System, West Haven, Connecticut; 115 Department of Medicine (Digestive Diseases), Yale University School of Medicine, New Haven, Connecticut

## Abstract

**Importance:**

The Colonoscopy Versus Fecal Immunochemical Test in Reducing Mortality From Colorectal Cancer (CONFIRM) randomized clinical trial sought to recruit 50 000 adults into a study comparing colorectal cancer (CRC) mortality outcomes after randomization to either an annual fecal immunochemical test (FIT) or colonoscopy.

**Objective:**

To (1) describe study participant characteristics and (2) examine who declined participation because of a preference for colonoscopy or stool testing (ie, fecal occult blood test [FOBT]/FIT) and assess that preference’s association with geographic and temporal factors.

**Design, Setting, and Participants:**

This cross-sectional study within CONFIRM, which completed enrollment through 46 Department of Veterans Affairs medical centers between May 22, 2012, and December 1, 2017, with follow-up planned through 2028, comprised veterans aged 50 to 75 years with an average CRC risk and due for screening. Data were analyzed between March 7 and December 5, 2022.

**Exposure:**

Case report forms were used to capture enrolled participant data and reasons for declining participation among otherwise eligible individuals.

**Main Outcomes and Measures:**

Descriptive statistics were used to characterize the cohort overall and by intervention. Among individuals declining participation, logistic regression was used to compare preference for FOBT/FIT or colonoscopy by recruitment region and year.

**Results:**

A total of 50 126 participants were recruited (mean [SD] age, 59.1 [6.9] years; 46 618 [93.0%] male and 3508 [7.0%] female). The cohort was racially and ethnically diverse, with 748 (1.5%) identifying as Asian, 12 021 (24.0%) as Black, 415 (0.8%) as Native American or Alaska Native, 34 629 (69.1%) as White, and 1877 (3.7%) as other race, including multiracial; and 5734 (11.4%) as having Hispanic ethnicity. Of the 11 109 eligible individuals who declined participation (18.0%), 4824 (43.4%) declined due to a stated preference for a specific screening test, with FOBT/FIT being the most preferred method (2820 [58.5%]) vs colonoscopy (1958 [40.6%]; *P* < .001) or other screening tests (46 [1.0%] *P* < .001). Preference for FOBT/FIT was strongest in the West (963 of 1472 [65.4%]) and modest elsewhere, ranging from 199 of 371 (53.6%) in the Northeast to 884 of 1543 (57.3%) in the Midwest (*P* = .001). Adjusting for region, the preference for FOBT/FIT increased by 19% per recruitment year (odds ratio, 1.19; 95% CI, 1.14-1.25).

**Conclusions and Relevance:**

In this cross-sectional analysis of veterans choosing nonenrollment in the CONFIRM study, those who declined participation more often preferred FOBT or FIT over colonoscopy. This preference increased over time and was strongest in the western US and may provide insight into trends in CRC screening preferences.

## Key Points

**Question:**

What are the characteristics of US veterans enrolling or declining participation in the Colonoscopy Versus Fecal Immunochemical Test in Reducing Mortality From Colorectal Cancer (CONFIRM) study?

**Findings:**

This cross-sectional study in 50 126 predominantly male and racially and ethnically diverse veterans at average risk for colorectal cancer found that declining participation in CONFIRM was associated with a preference for stool testing over colonoscopy. This preference increased over the recruitment period and was more frequent in the western US.

**Meaning:**

These trends in stool testing preference vs colonoscopy may provide insight into national screening trends in the US.

## Teaser

This cross-sectional study assesses reasons for nonparticipation in a colorectal cancer (CRC) screening clinical trial and their association with geographic and temporal factors.

## Introduction

Colorectal cancer (CRC) is the second leading cause of cancer death in the US.^1^ Screening has been shown to significantly reduce both CRC incidence and mortality and is widely recommended by the US Preventive Services Task Force, American Cancer Society, and US Multi-Society Task Force on Colorectal Cancer.^2,3,4,5,6^ While CRC screening is widely recommended, there is no consensus on a single best option for screening. The US Preventive Services Task Force^5^ and American Cancer Society^6^ recommend equally a panel of up to 6 options for screening. The Multi-Society Task Force on Colorectal Cancer also includes a similar panel of options but places 2 of the tests (colonoscopy and fecal immunochemical test [FIT]) as the most highly recommended approaches.^3,4^

Given that there is currently a panel of options for CRC screening, there remains interest in determining which test is most effective in preventing CRC and death from CRC. Colonoscopy is the most widely used test in the US for CRC screening.^7,8^ Colonoscopy affords several advantages relative to the other screening tests, including direct evaluation of the entire colonic mucosa and the opportunity to simultaneously remove colorectal polyps. Moreover, colonoscopy is required for direct colonic evaluation when other screening tests are abnormal. However, colonoscopy is also the most invasive test option, and bleeding and colonic perforation are major complications from the procedure.^9^ Often, the test is performed with at least moderate sedation and requires a complete bowel preparation. Given these downsides, there remains interest in less invasive approaches for screening.

The examination of stool for occult blood has long been used as a screening tool,^10^ and there is evidence from randomized clinical trials to support its use.^11^ Fecal immunochemical testing is a direct measure of hemoglobin in stool and is increasingly used both globally^12^ and within organized US screening programs.^13^ While tests like FIT are noninvasive, they do have some downsides relative to colonoscopy. The one-time sensitivity of FIT for cancer and cancer precursors^14^ is significantly less than one-time colonoscopy, although modeling studies have suggested that a program of FIT screening may achieve similar outcomes.^15^

The Colonoscopy Versus Fecal Immunochemical Test in Reducing Mortality From Colorectal Cancer (CONFIRM)^16^ study is a randomized trial that directly compares colonoscopy with FIT for the prevention of CRC mortality. The CONFIRM study is unique as it is the only large-scale comparative effectiveness study of CRC screening in the US and uses CRC mortality as a primary outcome. In this report, we describe the baseline characteristics of the enrolled cohort and explore reasons for ineligibility and nonparticipation in individuals who were eligible for study but declined enrollment.

## Methods

### Study Participants

This cross-sectional study uses data from the CONFIRM study. Full details of the trial design have been previously published.^16^ In brief, CONFIRM is a pragmatic, prospective, randomized, controlled, superiority trial comparing the effectiveness of screening colonoscopy and annual FIT in veterans at average risk for CRC, with a primary end point of CRC mortality. The CONFIRM study is approved by the Department of Veterans Affairs (VA) Central Institutional Review Board (CIRB). An external data monitoring committee reviews the trial semiannually and has access to unmasked outcome data. For individuals eligible and willing to participate, written informed consent was obtained by the coordinator. Consent was not obtained from nonparticipants, and no personal identifying information was retained for those individuals. While the population for the analysis is based on a randomized clinical trial, the data presented here are cross-sectional, drawn from baseline survey information from participants or potential participants; thus, this study uses the Strengthening the Reporting of Observational Studies in Epidemiology (STROBE) reporting guideline.

Participant recruitment occurred across 46 VA medical centers between May 22, 2012, and December 1, 2017, with planned follow-up through 2028. Veterans aged 50 to 75 years without signs or symptoms of CRC and due for screening were eligible for enrollment. Prior screening test use was allowed but had to be outside the screening window associated with a specific modality (eg, no exposure to colonoscopy in the past 9.5 years). Various recruitment efforts were used, including outreach to health care practitioners through on-site study coordinators and posters and mailed outreach to veterans who appeared to meet eligibility criteria. Manual or automated medical record review processes leveraging administrative codes and/or electronic screening reminder flags were performed to identify veterans who appeared due for screening. Prior to any direct contact with the veteran, VA CIRB-approved opt-out letters were used that also allowed the veteran to contact the coordinator directly if they were interested in study participation.

Veterans appearing eligible (through initial review) and either expressing interest in study participation or not opting out of contact were interviewed for eligibility and interest either in person or by telephone. Study coordinators were instructed to document this initial eligibility screening for those veterans who were directly interviewed (ie, fully screened). However, documentation was not retained for the many individuals whose medical records were screened for eligibility or from those who did not complete the brief screening interview (eg, quickly declined research participation when reached by phone). For veterans deemed eligible to participate, the coordinator sought to enroll the individual. When eligible individuals chose not to enroll, the coordinator indicated the reasons given for declining participation. The coordinator captured both the determination of eligibility and reasons for declining enrollment using a standardized data entry case report form, and multiple reasons could be cited for a single individual (eAppendix 1 in Supplement 1).

### Assessment of Baseline Characteristics

Enrolled participants completed a baseline questionnaire including demographic characteristics, medication use (eg, aspirin, nonsteroidal anti-inflammatory drugs, statins), use of substances (eg, tobacco, alcohol), exercise, any prior CRC screening, and family history of CRC (eAppendix 2 in Supplement 1). Race and ethnicity were self-reported by study participants. This information was collected and reported here to understand the generalizability of our study results to the US population at large and to assess the association of these factors with CRC outcomes. Female participants were queried with an additional survey, including questions about parity, medications (eg, hormones), and other exposures (eg, prior oophorectomy) that could modify CRC risk (eAppendix 3 in Supplement 1). After completing the baseline assessment, participants were randomized 1:1 to either colonoscopy or annual FIT screening, with concealed allocation and stratification by medical center using a random permuted block scheme with variable block size.

### Statistical Analysis

Baseline data are summarized as number (percentage), mean (SD), or median (IQR), as appropriate. Reasons for nonenrollment are summarized for veterans who were determined to be ineligible for the study or who were eligible but subsequently declined participation. Given that FIT and colonoscopy are the 2 CRC screening interventions under study in CONFIRM, we also explored associations between specific preferences for one of these tests over the other with recruitment region and year. Because these individuals declined participation, we had no information on their demographic or other characteristics. Descriptive statistics (ie, counts and percentages) were used to describe preference for FIT or colonoscopy across the region (Northeast, South, Midwest, or West) and calendar year (2012-2017) of recruitment, and the χ^2^ test was used to assess statistical significance across these factors. The linear association between screening preference and calendar year was assessed using the Cochran-Mantel-Haenszel test. The assignment of CONFIRM sites to region is shown in eFigure 1 in Supplement 1. Univariable logistic regression was then used to test the association between the covariates (recruitment region, study year) and the binary outcome of preference for FIT vs colonoscopy. Covariates with *P* < .10 from the univariable analysis were then included in a multivariable logistic regression. A 2-sided *P* < .05 was considered significant. Data were analyzed between March 7 and December 5, 2022, using SAS, version 9.4 statistical software (SAS Institute Inc).

## Results

**Figure 1.**
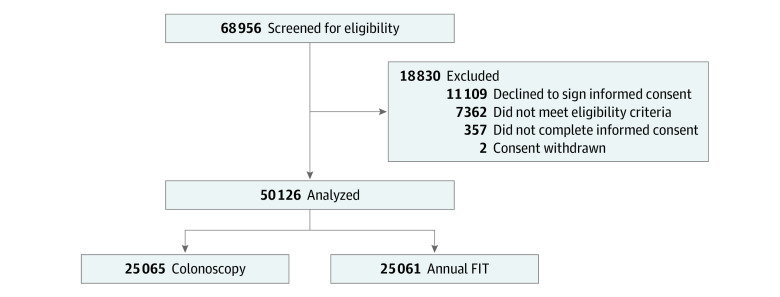
Flow Diagram Describing the Screened, Enrolled, and Randomized Colonoscopy Versus Fecal Immunochemical Test in Reducing Mortality From Colorectal Cancer Population FIT indicates fecal immunochemical testing.

During the enrollment phase, 50 126 participants were successfully recruited (mean [SD] age, 59.1 [6.9] years; male, 46 618 [93.0%]; female, 3508 [7.0%]). To accomplish recruitment, eligibility screening was documented and complete for 68 956 individuals, of whom 61 594 (89.2%) were found to be eligible for enrollment (Figure 1). After excluding those who either declined to provide informed consent (n = 11 109) or did not complete the process of informed consent (n = 357), 50 128 were initially randomized. Two of those participants were subsequently withdrawn when an audit revealed incomplete informed consent or Health Insurance Portability and Accountability Act documents on file, leaving 50 126 randomized participants.

### Baseline Characteristics of CONFIRM Participants

Study participants were enrolled from 46 VA medical centers, with a median of 1027 (range, 42-2760) participants per facility. Demographic and selected characteristics of the cohort are presented in Table 1. Age distribution was skewed toward younger veterans (17 117 [34.1%] were aged 50-54 years, while only 3610 [7.2%] were aged 70-75 years). A protocol deviation resulted in the recruitment of 13 veterans aged 45 to 49 years. After discussion with the VA CIRB, a decision was made to keep these veterans in the study (Table 1). With regard to race, 748 individuals (1.5%) self-identified as Asian, 12 021 (24.0%) as Black or African American, 415 (0.8%) as Native American or Alaska Native, 34 629 (69.1%) as White, and 1877 (3.7%) as other (including multiracial). With regard to ethnicity, 5734 (11.4%) self-identified as Spanish, Hispanic, or Latino.

**Table 1.** Description of Colonoscopy vs Fecal Immunochemical Test in Reducing Mortality From Colorectal Cancer Study Participants^a^

Characteristic	No. (%)			
	Entire cohort	FIT arm	Colonoscopy arm
No. of participants	50 126	25 061 (50.0)	25 065 (50.0)
Age at randomization, y			
45-49	13 (<1)	7 (<1)	6 (<1)
50-54	17 117 (34.1)	8566 (34.2)	8551 (34.1)
55-59	9155 (18.3)	4528 (18.1)	4627 (18.5)
60-64	10 328 (20.6)	5189 (20.7)	5139 (20.5)
65-69	9903 (19.8)	4967 (19.8)	4936 (19.7)
70-75	3610 (7.2)	1804(7.2)	1806 (7.2)
Sex			
Female	3508 (7.0)	1799 (7.2)	1709 (6.8)
Male	46 618 (93.0)	23 262 (92.8)	23 356 (93.2)
Race			
Asian	748 (1.5)	348 (1.4)	400 (1.6)
Black or African American	12 021 (24.0)	6056 (24.2)	5965 (23.8)
Native American or Alaska Native	415 (0.8)	209 (0.8)	206 (0.8)
White	34 629 (69.1)	17 290 (69.0)	17 339 (69.2)
Other (including multiracial)^b^	1877 (3.7)	947 (3.8)	930 (3.7)
Ethnicity			
Hispanic^c^	5734 (11.4)	2913 (11.6)	2821 (11.3)
Not Hispanic	44 254 (88.3)	22 086 (88.1)	22 168 (88.4)
Education			
High school or less	14 097 (28.1)	7058 (28.2)	7039 (28.1)
Some college	17 631 (35.2)	8796 (35.1)	8835 (35.2)
College degree or higher	18 193 (36.3)	9099 (36.3)	9094 (36.3)
BMI, mean (SD)	30.2 (5.9)	30.2 (5.8)	30.2 (5.9)
Smoking			
Never	18 604 (37.1)	9241 (36.9)	9363 (37.4)
Ever	31 474 (62.8)	15 800 (63.0)	15 674 (62.5)
Current	12 682 (40.3)^d^	6325 (40.0)^d^	6357 (40.6)^d^
Former	18 792 (59.7)^d^	9475 (60.0)^d^	9317 (59.4)^d^
Alcohol, drinks/d, mean (SD)	0.78 (1.92)	0.78 (1.97)	0.78 (1.87)
≥1 Second-degree relative with CRC, yes	1924 (3.8)	981 (3.9)	943 (3.8)
Medications (current use)			
Multivitamin	19 946 (39.8)	9662 (39.8)	9984 (39.8)
Calcium supplement	6412 (12.8)	3168 (12.6)	3244 (12.9)
Vitamin D	12 366 (24.7)	6133 (24.5)	6233 (24.9)
Statin	19 565 (39.0)	9823 (39.2)	9742 (38.9)
Aspirin	20 666 (41.2)	10 327 (41.2)	10 339 (41.3)
Nonsteroidal anti-inflammatory drug	15 992 (31.9)	7961 (31.8)	8031 (32.0)
Hormone replacement therapy (women only)	230 (6.6)	113 (6.3)	117 (6.9)
Military service type			
Active duty	49 335 (98.4)	24 665 (98.4)	24 670 (98.4)
Reserves only	747 (1.5)	372 (1.5)	375 (1.5)

Abbreviations: BMI, body mass index (calculated as weight in kilograms divided by height in meters squared); CRC, colorectal cancer; FIT, fecal immunochemical testing.

^a^ Percentages for each variable may not sum to 100% because of refusal or missing data.

^b^ No breakdown of the races and ethnicities that made up the other category was available.

^c^ Spanish, Hispanic, or Latino.

^d^ Percentage calculated with the denominator of ever-smokers.

Other characteristics of the population included a mean (SD) body mass index (as measured by weight in kilograms divided by height in meters squared) of 30.2 (5.9). Most of the participants reported having at least some post–high school education (35 824 [71.5%]). Some history of smoking was common (31 474 [62.8%]), with current smoking reported by 12 682 (40.3%).

Aspirin use was reported by 20 666 participants (41.2%), while statin use was reported by 19 565 (39.0%). Among the 3507 participants who completed the dedicated baseline survey for women, 230 (6.6%) reported current hormone replacement therapy. As would be expected in a randomized study of this size, characteristics were well balanced between the 2 intervention groups.

### Reasons for Study Ineligibility

Of individuals fully screened, 7362 (10.7%) were deemed ineligible, and a total of 8240 reasons were documented for ineligibility (eTable 1 in Supplement 1). The most common reason for ineligibility was that the veteran was not due for screening (4149 [50.4%] across all reasons). This was due most frequently to prior exposure to colonoscopy (3288 [39.9%]) and much less commonly to other screening tests. Ineligibility for average-risk screening because of some other personal or family history also was commonly encountered (2081 [25.3%] of all reasons). For example, having a first-degree family history of CRC (930 [11.3%]) was a common reason. Signs and symptoms of CRC (836 [10.1%]) and research participation considerations (979 [11.9%]) were also often cited as reasons for ineligibility. For example, given the potential need to send annual FIT kits over the long time frame of the study, a determination by the coordinator that study personnel would not be able to contact an individual over time (eg, due to unstable housing or lack of a permanent mailing address) was a reason for ineligibility (764 [9.3%]).

### Reasons for Declining Enrollment

Among initially eligible veterans, 11 109 declined enrollment, of whom 5037 (45.3%) did not provide a specific reason (Table 2). For those providing a specific reason (n = 6072), most often the veteran preferred a particular screening test (4824 [79.4%]). Concerns about participating in research were cited by a small number of individuals, including factors such as participant burden (n = 411) and privacy and confidentiality (n = 44).

**Table 2.** Summary of Specific Reasons for Individuals Declining Participation (n = 11 109)

Specified reason	No. (%) declining to participate (n = 6072)^a^
Prefers screening with a specific modality	
Any	4824 (79.4)
FOBT/FIT	2820 (58.4)^b^
Colonoscopy	1958 (40.6)^b^
Flexible sigmoidoscopy	14 (0.3)^b^
CT colonography	11 (0.2)^b^
Stool DNA	8 (0.2)^b^
Other screening test	13 (0.3)^b^
Not interested in colorectal cancer screening	793 (13.0)
Concerns regarding research participation	
Any	455 (9.4)
Participant burden (including follow-up and surveys)	411 (90.3)^c^
Privacy, confidentiality, use of Social Security Number	44 (9.7)^c^

Abbreviations: CT, computed tomography; FIT, fecal immunochemical test; FOBT, fecal occult blood test.

^a^ A total of 5037 individuals did not provide, refused to provide, or offered some other reason for nonparticipation.

^b^ Proportion of individuals with a preference for a specific screening modality.

^c^ Proportion of individuals with concerns regarding research participation.

### Analysis of Veterans Declining Study Participation Because of Preference for FIT or Colonoscopy

**Figure 2.**
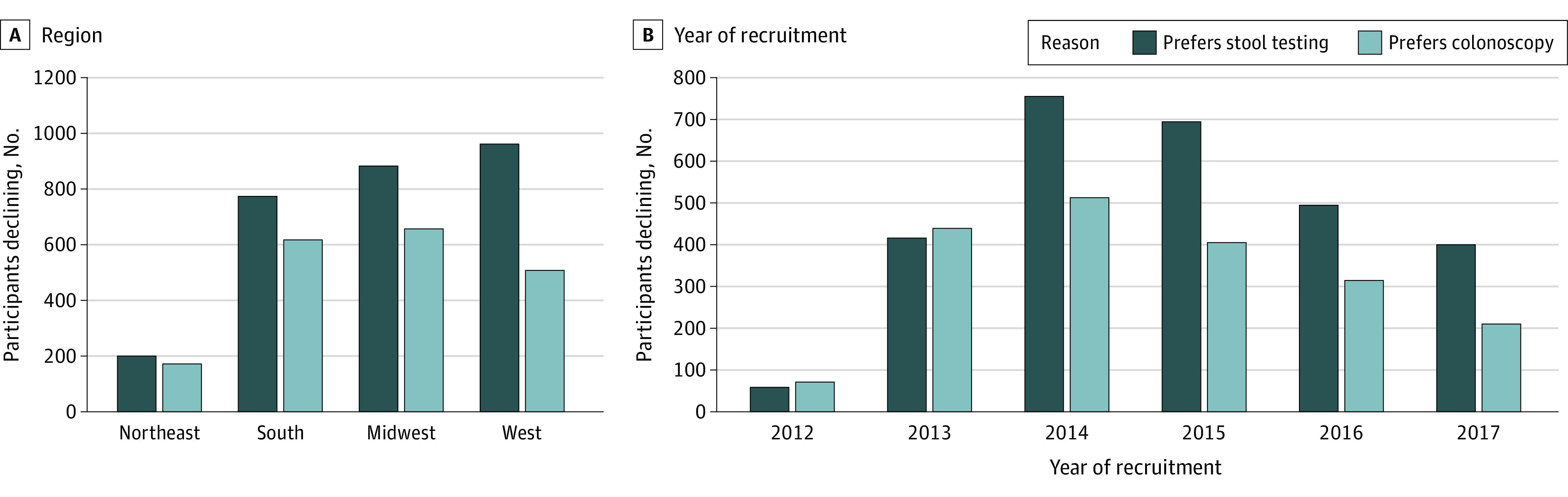
Individuals Eligible for Participation Who Declined Because of a Stated Preference for Stool Testing With Fecal Occult Blood Test or Fecal Immunochemical Test vs Colonoscopy, Stratified by Region and Year of Attempted Recruitment

Among veterans declining participation because of a preference for a specific screening test (n = 4824), more declined because of a preference for fecal occult blood test (FOBT)/FIT (2820 [58.4%]) than for colonoscopy (1958 [40.6%]; *P* < .001). Preference for FOBT/FIT varied by region and was strongest in the West (963 of 1472 [65.4%]) and more modest in the other areas of the country (884 of 1543 [57.3%] in the Midwest, 774 of 1392 [55.6%] in the South, and 199 of 371 [53.6%] in the Northeast; *P* < .001) (Figure 2A; eTable 2 in Supplement 1). Preference for FOBT/FIT also increased over recruitment years from 59 of 131 (45.0%) in 2012 to 400 of 611 (65.5%) in 2017 as shown in Figure 2B.

The results of the modeling describing the association of region and year of recruitment with preference for stool testing with FOBT/FIT are shown in Table 3. Among veterans declining participation, the odds of preference for stool testing with FOBT/FIT increased by 19% per year (odds ratio, 1.19; 95% CI, 1.14-1.25). Preference for FOBT/FIT was strongest in the West relative to all other regions.

**Table 3.** Univariable and Multivariable Association of Study Site Region and Year of Recruitment With Preference for Stool Testing With Fecal Occult Blood Test or Fecal Immunochemical Test vs Colonoscopy Among Individuals Declining Enrollment (n = 4778)

Analysis type and effect	OR (95% CI)
Univariable	
Midwest vs West	0.71 (0.61-0.82)
Northeast vs West	0.61 (0.49-0.77)
South vs West	0.66 (0.57-0.77)
Study year	1.17 (1.12-1.22)
Multivariable	
Midwest vs West	0.73(0.63-0.85)
Northeast vs West	0.56 (0.45-0.71)
South vs West	0.61 (0.52-0.71)
Study Year	1.19 (1.14-1.25)

Abbreviation: OR, odds ratio.

Notably, between 2012 and 2017, 6 participating VA facilities ceased enrollment of new participants because of low recruitment. Preferences by region and year of recruitment are presented for all sites and separately, excluding sites that discontinued recruitment prior to the end of the recruitment period, and the observed patterns were similar (eFigures 2 and 3 in Supplement 1).

## Discussion

The CONFIRM study has enrolled 50 126 veterans at average risk for CRC and randomized them to screening with either colonoscopy or annual FIT. In this cross-sectional study, we describe characteristics of the cohort and factors associated with ineligibility for enrollment or declining participation in the study. We specifically analyzed data from individuals who declined because of a stated preference for screening with FIT or colonoscopy and found that preference varied by region of recruitment and increased over the period of recruitment.

A major design goal of the CONFIRM study was to recruit a diverse cohort of individuals representative of the US population. While the cohort is largely male, it is diverse with respect to race and ethnicity. Given that recruitment was within the VA health care system, it was not possible to have strong female participation, despite adjunctive efforts to recruit female veterans. As of 2017, it was estimated that approximately 9.6% of VA health system users were female,^17^ and CONFIRM recruited slightly less than that. This lower average recruitment in female veterans may be due to a lower median age for female veterans using VA services (48 years for women and 64 years for men),^17^ so many would not be eligible for CONFIRM. With regard to race, the CONFIRM cohort is diverse with respect to the recruitment of individuals identifying as Black or African American. According to the most recent US census estimates, 13.4% of the US population identifies as Black or African American,^18^ and these individuals represent one-quarter of the CONFIRM cohort. Recruitment of other racial and ethnic groups was somewhat less successful. For example, US census data indicate that 18.5% of the population identifies as Hispanic or Latino,^18^ but 11.4% of the CONFIRM cohort self-reported as Spanish, Hispanic, or Latino. The CONFIRM study is more representative of US populations relative to prior US screening trials. For example, in a large, randomized US trial of flexible sigmoidoscopy,^19^ 85% of participants were White (vs CONFIRM at 69.1%) and 1.8% Hispanic or Latino (vs CONFIRM at 11.4%).

Accomplishing racial and ethnic diversity within large clinical trials is often challenging. For example, 1 analysis examining racial and ethnic distribution in oncology trials (N = 145) showed that Black individuals comprised only 22% and Hispanic individuals 44% of the expected proportion.^20^ Within the framework of CONFIRM, accomplishing diversity within the population under study is important because CRC outcomes differ across some subgroups. Colorectal cancer incidence and mortality is higher for Black individuals relative to White and Asian or Pacific Islander individuals according to the most recent US statistics.^1^ A number of factors within CONFIRM facilitated broad-based recruitment with regard to these factors. First, many VA sites participated (N = 46), and geographically, they were located across the entire US, including Hawaii and Puerto Rico. Second, the effect of socioeconomic status, a well-recognized barrier to CRC screening,^21^ is largely mitigated in the VA health system. There is good evidence that screening uptake in the VA is as good, if not better, in racial and ethnic minority individuals relative to White individuals.^22^ In addition, colonoscopy (as a study intervention) could be costly to those without insurance, which might affect recruitment in non-VA studies. However, barriers to care for VA-eligible veterans are reduced, as all study examinations were completed as part of routine care and all co-pays (eg, preparation for study colonoscopy) were waived entirely for study participants. There is evidence that waiver of copayments for colonoscopy can increase screening participation.^23^ Participants would still require someone to drive them home after receiving sedation for colonoscopy.

While this report describes trial participant data in detail, we also were able to explore reasons for nonparticipation in CONFIRM. In terms of veterans found ineligible for enrollment, the most common reason was that the individual was not due for screening. This finding is not particularly surprising given that approximately 80% of veterans enrolled in primary care within the VA are found to be up to date with CRC screening.^22^ However, the protocol did allow recruitment of those with prior screening test use. Certainly, over the course of recruitment, some individuals initially found to be ineligible (eg, because of a recent stool test) subsequently became eligible (eg, once they were >10 months past their last FIT) and were enrolled.

We were also interested in studying veterans who were eligible for enrollment but declined participation. The most common reason for declining was that the individual simply preferred one of the screening tests and did not want to be randomly assigned. This observed variation in preference for screening between the 2 most common screening options is, in and of itself, an important research finding. Interestingly, within this VA-based cohort, preference was higher for FIT than colonoscopy in those declining participation. This finding may reflect long-standing patterns of screening test use in the VA. In 2003, estimates using VA data suggested that 90% of CRC screening was done by FOBT.^24^ A more recent analysis suggested some modification of this pattern with more colonoscopy use in the VA, with nearly one-third screened by colonoscopy.^25^ Nonetheless, this pattern of screening test use differs sharply from the US at large, where colonoscopy is the dominant modality and stool testing is rarely used.^7^ But preferences for noninvasive screening with FOBT have also been found in non-VA settings. In a randomized study of community clinics in the San Francisco area, Inadomi et al^26^ demonstrated that offering screening colonoscopy results in lower participation than offering either FOBT or a choice of colonoscopy or FOBT. Furthermore, when given a choice, the preference for FOBT or colonoscopy varied by race, with a greater preference for FOBT among African American individuals. As noted earlier, current decision models estimate similar effectiveness for colonoscopy and annual FIT screening, and this is the foundation for the equipoise of randomization in CONFIRM. However, if one of the screening tests is demonstrated to be superior to the other for reducing CRC mortality, preferences for CRC screening tests may be dramatically altered.

We also observed time trends for increasing FIT preference over the recruitment period and some regional preference for FIT. Specifically, we observed a greater preference for FIT in the western US, and this finding would be consistent with recently reported National Health Interview Survey data that examined screening trends in 2019 and 2012.^27^ Anecdotally, we have observed that the CRC screening culture seems to vary at different VA facilities around the US, as some VA medical centers have adopted a colonoscopy-first approach while others have encouraged FIT screening. The literature examining screening preference, including direct comparison of preference for stool-based testing or colonoscopy, is complex. Not surprisingly, investigators using analytic hierarchy processes that deconstruct the higher-level decision (ie, which test to choose) from simpler criteria (eg, whether an individual values test accuracy vs convenience) have found colonoscopy to be the preferred test by individuals most valuing accuracy.^28^ Recently, though, a nationwide survey of US adults aged 50 years or older using conjoint analysis found significantly higher preference for annual FIT (77.4%) relative to colonoscopy every 10 years (22.6%; *P* = .004).^29^ The regional variation we observed may partly be explained by other factors that we could not directly measure. For example, some studies have found associations of test preference with socioeconomic factors. In one study among veterans (N = 2068) a multivariate analysis showed that those with incomes of $20 000 to $40 000 were more likely to prefer colonoscopy relative to those with incomes of $20 000 or less (odds ratio, 1.46; 95% CI, 1.11-1.91).^30^

### Strengths and Limitations

Strengths of our analysis examining preference for colonoscopy or FIT among veterans declining participation in CONFIRM include a large sample of enrollees from across the US. Furthermore, this information was gathered directly from the individuals during a structured conversation with one of our coordinators that was guided by a dedicated case report form designed for the purpose of understanding both eligibility for enrollment and declining participation.

Limitations of our analysis include some lack of generalizability since the work was performed entirely within veterans whose preferences for screening may not be generally representative of US citizens at large. In addition, CONFIRM was not performed in a random sample of VA medical centers. To maximize recruitment, we targeted centers using high numbers of stool screening tests and avoided those with a colonoscopy-first approach where we believed recruitment would be challenging. Such factors may influence the opinions of the veterans receiving care at those medical centers. Moreover, as we described, some of the temporal changes we observed in preference for FIT may be partly related to features of recruitment itself. For example, we discontinued recruitment at centers where recruitment was going poorly, and preference for colonoscopy may have been associated with poor recruitment efforts. It is also conceivable that coordinators were approaching veterans with a history of stool-based screening more frequently at the end of recruitment as those individuals would be coming due more frequently, whereas a history of colonoscopy had a much longer exclusion period. There is good evidence that history of use of a particular CRC screening test is associated with subsequent preference for that screening test.^31^ Finally, as noted, we do not have detailed information on individual characteristics (eg, age and race) of veterans declining participation.

## Conclusions

The findings of this cross-sectional analysis of the recruited and eligible population for the CONFIRM study provide important insight into both groups. The CONFIRM study recruited its cohort of 50 126 adults with an average risk for CRC to compare the effectiveness of colonoscopy vs annual FIT screening for the reduction of CRC mortality. The recruited population better approximates the diversity of the US population in terms of race and ethnicity than prior large screening studies, and this is the only US study to our knowledge currently comparing these common CRC screening tests. Separately, we were able to evaluate reasons why individuals chose not to participate, which helps to elucidate current trends in preferences for noninvasive CRC screening. Specifically, our study suggests some increase in the preference for noninvasive FIT over time, especially in certain regions of the country. Further work to better understand contemporary changes in preference for CRC screening in the US is warranted.

## Notes

Supplement 1. **eTable 1.** Summary of Reasons for Ineligibility During the Screening Process (n = 7362) **eTable 2.** Predictors of Preference for Stool Testing by FOBT/FIT Relative to Colonoscopy **eFigure 1.** Map Demonstrating Sites Participating in CONFIRM Recruitment **eFigure 2.** Trends in Those Declining Participation by Year and Region—Including All Sites **eFigure 3.** Trends in Those Declining Participation by Year and Region—Excluding Sites That Ceased Recruitment During Recruitment Phase **eAppendix 1.** Screening/Eligibility Form **eAppendix 2.** Baseline Data Collection Form **eAppendix 3.** Baseline Data Collection Form, Females Only

Supplement 2.
Nonauthor Collaborators

Supplement 3.
Data Sharing Statement
