# Error in Byline

**DOI:** 10.1001/jamanetworkopen.2023.30304

**Published:** 2023-08-11

In the Original Investigation titled “Baseline Features and Reasons for Nonparticipation in the Colonoscopy Versus Fecal Immunochemical Test in Reducing Mortality From Colorectal Cancer (CONFIRM) Study, a Colorectal Cancer Screening Trial,”^1^ published July 11, 2023, author Barbara Del Curto’s academic degrees were incorrect in the byline; her correct degree is a BS. This article has been corrected.^1^
