# Supplementary material for: Baseline Features and Reasons for Nonparticipation in the Colonoscopy Versus Fecal Immunochemical Test in Reducing Mortality From Colorectal Cancer (CONFIRM) Study, a Colorectal Cancer Screening Trial
Source: JAMA Netw Open. 2023 Jul 11;6(7):e2321730. doi: 10.1001/jamanetworkopen.2023.21730 (PMC10336619; doi:10.1001/jamanetworkopen.2023.21730)
Supplement: Supplement 1. — eTable 1. Summary of Reasons for Ineligibility During the Screening Process (n = 7362) eTable 2. Predictors of Preference for Stool Testing by FOBT/FIT Relative to Colonoscopy eFigure 1. Map Demonstrating Sites Participating in CONFIRM Recruitment eFigure 2. Trends in Those Declining Participation by Year and Region—Including All Sites eFigure 3. Trends in Those Declining Participation by Year and Region—Excluding Sites That Ceased Recruitment During Recruitment Phase eAppendix 1. Screening/Eligibility Form eAppendix 2. Baseline Data Collection Form eAppendix 3. Baseline Data Collection Form, Females Only [file jamanetwopen-e2321730-s001.pdf]

Supplemental Online Content

Robertson DJ, Dominitz JA, Beed A, et al; CONFIRM Study Group. Baseline features and reasons for nonparticipation in the Colonoscopy Versus Fecal Immunochemical Test in Reducing Mortality From Colorectal Cancer (CONFIRM) study, a colorectal cancer screening trial. *JAMA Netw Open*. 2023;6(7):e2321730. doi:10.1001/jamanetworkopen.2023.21730

eTable 1. Summary of Reasons for Ineligibility During the Screening Process (n = 7362)

eTable 2. Predictors of Preference for Stool Testing by FOBT/FIT Relative to Colonscopy

eFigure 1. Map Demonstrating Sites Participating in CONFIRM Recruitment

eFigure 2. Trends in Those Declining Participation by Year and Region—Including All Sites

eFigure 3. Trends in Those Declining Participation by Year and Region—Excluding Sites That Ceased Recruitment During Recruitment Phase

eAppendix 1. Screening/Eligibility Form

eAppendix 2. Baseline Data Collection Form

eAppendix 3. Baseline Data Collection Form, Females Only

This supplemental material has been provided by the authors to give readers additional information about their work.

eTable 1: Summary of Reasons for Ineligibility During the Screening Process (N=7362)

Reasons for Screen Failure	N* (8240)
Not due for screening	4149 (50.4%)
Colonoscopy within the past 9.5 years	3288
FOBT, FIT within past year; FIT-DNA within 3 years	722
Flexible Sigmoidoscopy within 5 years	84
CT Colonography within 5 years	38
Barium Enema within 5 years	17
Not eligible for average risk screening	2081 (25.3%)
Family history CRC (1st degree relative)	930
Significant co-morbidity excluding benefit from screening	656
Personal history of adenomatous polyps	331
Personal history of inflammatory bowel disease	52
Personal history of CRC	30
Pregnant	10
Age <50 or >75	72
Signs or symptoms of CRC	836 (10.1%)
>1 episode of rectal bleeding† in last 6 months	590
Iron deficiency anemia in last 6 months	174
Weight loss > 10% in last 6 months	72
Research participation considerations	979 (11.9%)
Likely unable to contact Veteran over time	764
Not able to provide informed consent	155
Already participating in another interventional study	49
Prisoner	11
Other	195 (2.4%)
Colonic resection	127
Not a Veteran	68

*Participants can be ineligible for multiple reasons and so the percent given is the percent of all reasons provided (N=8240)

†≥ 1 episode of bright red blood per rectum excluding only small amounts of blood on toilet tissue

FOBT , fecal occult blood test; FIT, fecal immunochemical test; CRC, colorectal cancer

© 2023 Robertson DJ et al. *JAMA Network Open*.

eTable 2: Predictors of Preference for Stool Testing by FOBT/FIT Relative to Colonoscopy

Comparator	Degrees of freedom	Wald Chi-square	P-Value
All regions	3	36.90	<0.0001
West vs. Northeast	1	17.33	<0.0001
West vs. South	1	27.80	<0.0001
West vs. Midwest	1	20.45	<0.0001
Northeast vs South	1	0.50	0.48
Northeast vs. Midwest	1	1.62	0.20
South vs. Midwest	1	0.76	0.38

© 2023 Robertson DJ et al. *JAMA Network Open.*

eFigure 1. Map Demonstrating Sites Participating in CONFIRM Recruitment

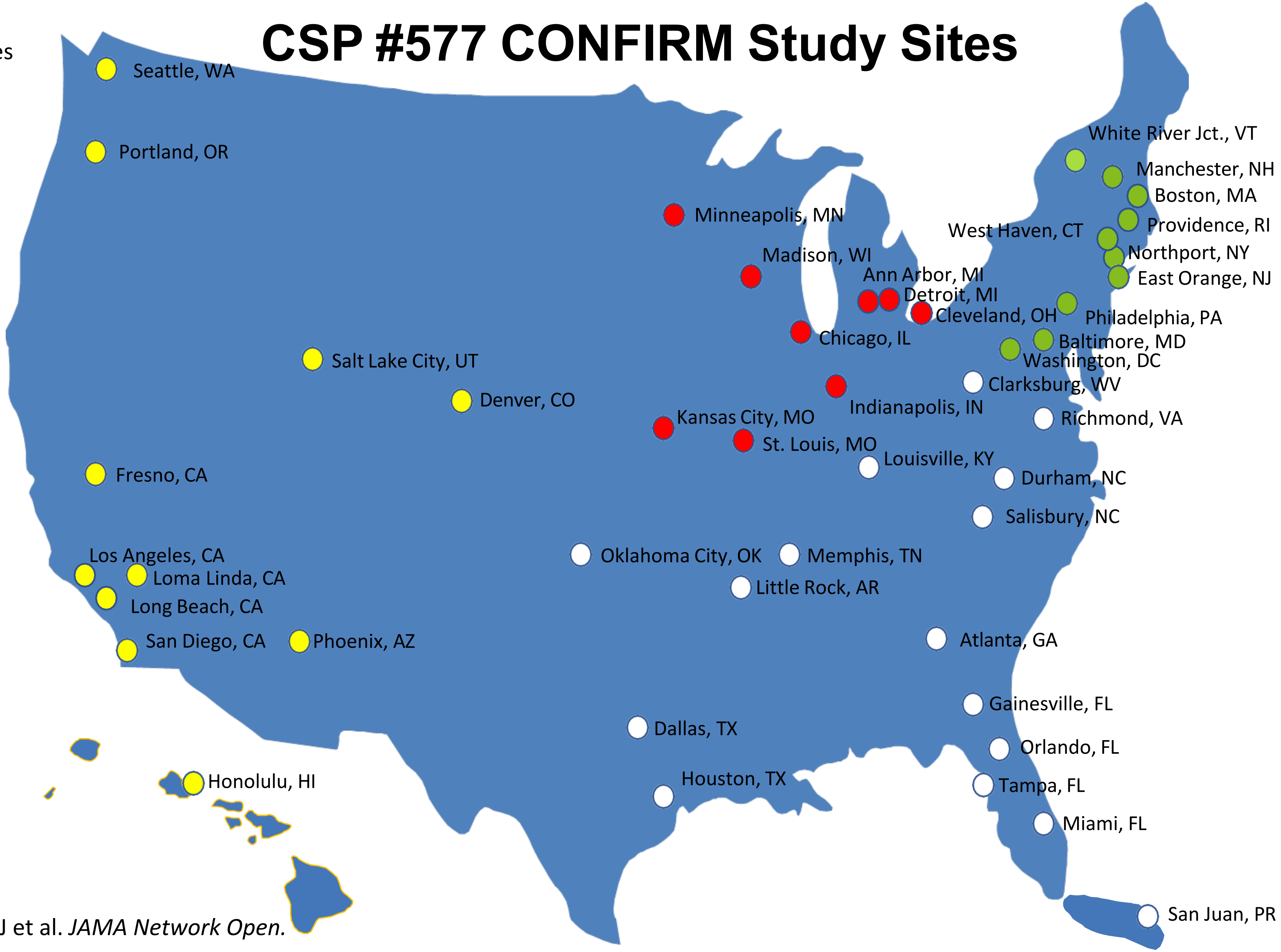

© 2023 Robertson DJ et al. *JAMA Network Open.*

eFigure 2. Trends in Those Declining Participation by Year and Region--Including All Sites

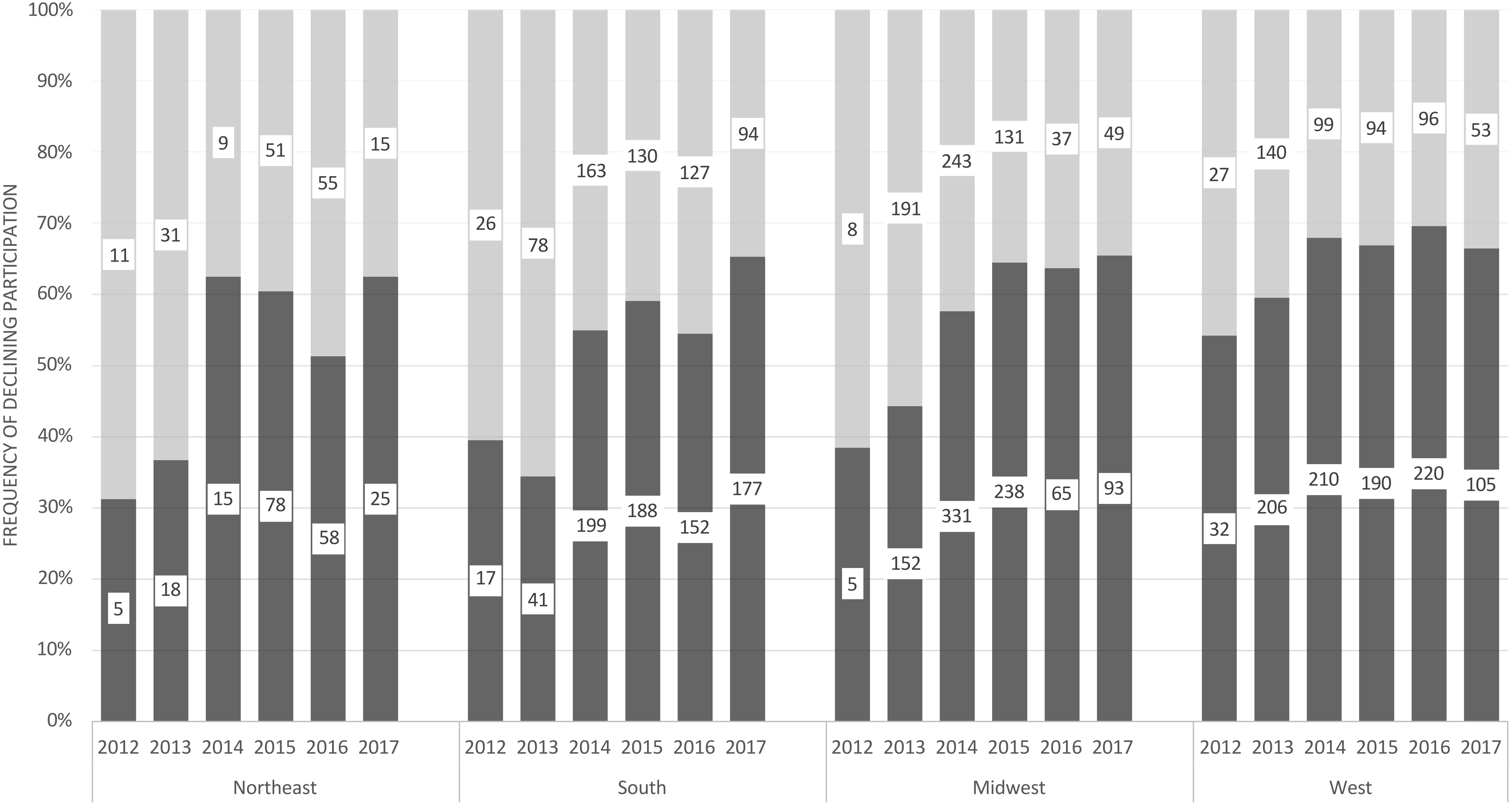

© 2023 Robertson DJ et al. *JAMA Network Open.*

eFigure 3. Trends in Those Declining Participation by Year and Region--Excluding Sites That Ceased Recruitment During Recruitment Phase

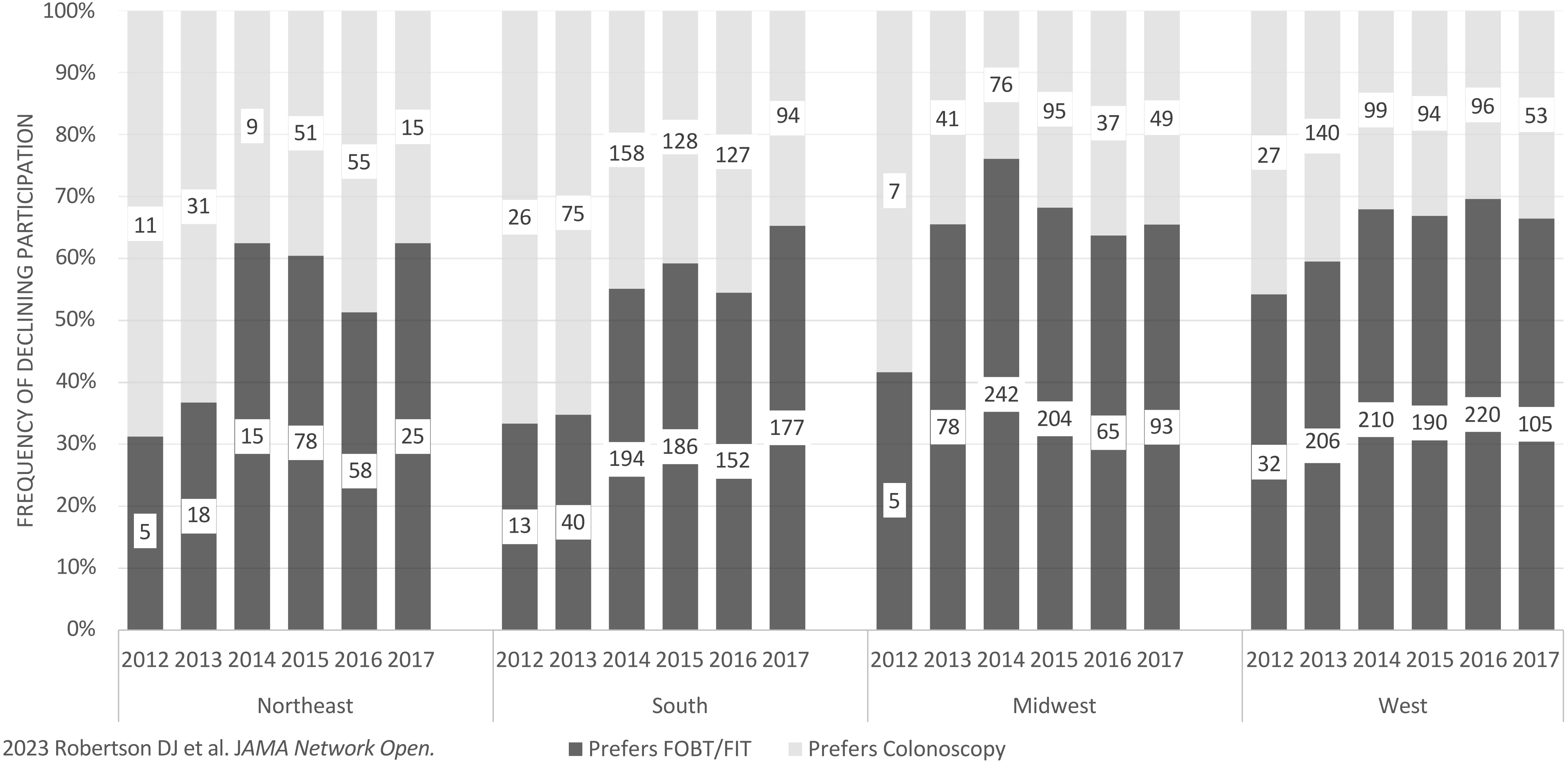

© 2023 Robertson DJ et al. *JAMA Network Open.*

0382225776

VA CSP #577

Colonoscopy vs. Fecal Immunochemical Test in Reducing Mortality from Colorectal Cancer (CONFIRM)

Form 01 - Screening/Eligibility Form

Site No.	ID No.	Date of Screening (mm/dd/yyyy)
☐☐	☐☐☐☐☐	☐☐ / ☐☐ / ☐☐☐☐

I. INCLUSION CRITERIA (ALL MUST BE "YES" IN ORDER TO BE ENROLLED)	Yes	No
A. Age >= 50 and <= 75 ?	☐	☐
B. Able to give informed consent?	☐	☐
C. Is a Veteran?	☐	☐

II. EXCLUSION CRITERIA (ALL MUST BE "NO" IN ORDER TO BE ENROLLED)	Yes	No
A. Any fecal occult blood test (FOBT) or fecal immunochemical test (FIT) within the past 10 months?	☐	☐
B. Colonoscopy within past 9.5 years?	☐	☐
C. Flexible sigmoidoscopy within past 5 years?	☐	☐
D. Barium Enema within past 5 years?	☐	☐
E. CT Colonography within past 5 years?	☐	☐
F. History of adenomatous polyps?	☐	☐
G. More than 1 episode of rectal bleeding within past 6 months?	☐	☐
H. Documented unexplained weight loss > = 10 % within the past 6 months?	☐	☐
I. Documented iron deficiency anemia within past 6 months?	☐	☐
J. Inflammatory Bowel disease (e.g., ulcerative colitis or Crohn's Disease)?	☐	☐
K. Colorectal Cancer?	☐	☐
L. Colonic Resection?	☐	☐
M. Significant comorbidity that would preclude benefit from screening or pose significant risk for performance of colonoscopy?	☐	☐
N. First degree relative diagnosed with colon or rectal cancer?	☐	☐
O. Pregnant?	☐	☐
P. Prisoner?	☐	☐
Q. Veteran in a concurrent interventional **colon** related study without CSP approval to co-enroll?	☐	☐
R. Likely inability to contact participant over time?	☐	☐

© 2023 Robertson DJ et al. *JAMA Network Open*.

4599225770

VA CSP #577
Colonoscopy vs. Fecal Immunochemical Test in Reducing Mortality from Colorectal Cancer (CONFIRM)
Form 01 - Screening/Eligibility Form

Site No. ☐☐ **ID No.** ☐☐☐☐☐ **Date of Screening (mm/dd/yyyy)** ☐☐ / ☐☐ / ☐☐☐☐

	Yes	No
**III. ELIGIBLE FOR CONSENT?**	☐	☐
**IV. SIGNED INFORMED CONSENTAND HIPAA?**	☐	☐
**V. WITHDREW INFORMED CONSENT PRIOR TO RANDOMIZATION?**	☐	☐

VI. REASON FOR NOT SIGNING INFORMED CONSENT OR REASON FOR WITHDRAWAL (check one):

- ☐ Prefers colonoscopy
- ☐ Prefers stool testing (FIT/FOBT)
- ☐ Prefers Flexible Sigmoidoscopy
- ☐ Prefers Barium Enema
- ☐ Prefers CT Colonography
- ☐ Prefers Stool DNA
- ☐ Prefers other screening modality, specify: ☐☐☐☐☐☐☐☐☐☐☐☐
- ☐ Not interested in having colorectal cancer screening
- ☐ Participant burden (travel, surveys, mailings, length of follow-up, etc)
- ☐ Concerns regarding privacy, confidentiality, use of SSN
- ☐ Participant refused to give a reason
- ☐ No show/unable to contact patient
- ☐ Patient changed mind about participating/not interested in participating.
- ☐ Other, specify: ☐☐☐☐☐☐☐☐☐☐☐☐☐☐☐☐☐☐☐

Staff Initials ☐☐☐

© 2023 Robertson DJ et al. *JAMA Network Open.*

2900430312

VA CSP #577
Colonoscopy vs.Fecal Immunochemical Test in Reducing Mortality from Colorectal Cancer
Form 02 - Baseline

Site No. ☐☐ **ID No.** ☐☐☐☐☐ **Date Form Completed (mm/dd/yyyy)** ☐☐ / ☐☐ / ☐☐☐☐

I. Demographics

A. Birthdate: ☐☐ / ☐☐ / ☐☐☐☐ (mm/dd/yyyy)

B. Sex: ☐ Male ☐ Female

C. Ethnicity - Spanish, Hispanic, Latino: **(check one)**

- ☐ No, not Spanish, Hispanic or Latino
- ☐ Yes, Mexican, Mexican American, Chicano
- ☐ Yes, Puerto Rican
- ☐ Yes, Cuban
- ☐ Yes, other Spanish, Hispanic or Latino
- ☐ Refused to answer

D. Race: **(check one or more)**

- ☐ White
- ☐ African-American or Black
- ☐ American Indian or Alaskan Native
- ☐ Native Hawaiian or Other Pacific Islander
- ☐ Chinese
- ☐ Japanese
- ☐ Asian Indian
- ☐ Filipino
- ☐ Other Asian
- ☐ Other, specify: ☐☐☐☐☐☐☐☐☐☐
- ☐ Refused to Answer

E. Education: **(check one)**

- ☐ Less than High School Diploma
- ☐ High School Diploma/GED
- ☐ Some College credit/but no Degree
- ☐ Associate Degree
- ☐ Bachelor's Degree
- ☐ Master's Degree
- ☐ Ph.D. or Professional Degree
- ☐ Refused to Answer

© 2023 Robertson DJ et al. *JAMA Network Open.*

VA CSP #577
Colonoscopy vs.Fecal Immunochemical Test in Reducing Mortality from Colorectal Cancer
Form 02 - Baseline

Site No.	ID No.	Date Form Completed (mm/dd/yyyy)
☐☐	☐☐☐☐☐	☐☐ / ☐☐ / ☐☐☐☐

II. Colonoscopy

A. Have you **EVER** had a colonoscopy?

☐ Yes ☐ No ☐ Don't Know

If yes,

1. How many times have you had a colonoscopy? ☐☐ times
2. How long ago was your last colonoscopy? ☐☐ . ☐☐ years

III. FOBT

A. Have you **EVER** had a fecal occult blood test (FOBT), such as a Hemoccult?

☐ Yes ☐ No ☐ Don't Know

If yes,

1. How many times have you had an FOBT? ☐☐ times
2. How long ago was your last FOBT? ☐☐ years ☐☐ months

IV. Flexible Sigmoidoscopy

A. Have you **EVER** had a flexible sigmoidoscopy?

☐ Yes ☐ No ☐ Don't Know

If yes,

1. How many times have you had a sigmoidoscopy? ☐☐ times
2. How long ago was your last sigmoidoscopy? ☐☐ . ☐☐ years

© 2023 Robertson DJ et al. *JAMA Network Open.*

Site No. ☐☐ **ID No.** ☐☐☐☐☐ **Date Form Completed (mm/dd/yyyy)** ☐☐ / ☐☐ / ☐☐☐☐

V. Barium Enema

A. Have you **EVER** had a barium enema?

☐ Yes ☐ No ☐ Don't Know

If yes,

1. How many times have you had a barium enema? ☐☐ times
2. How long ago was your last barium enema? ☐☐.☐☐ years

VI. CT Colonography

A. Have you **EVER** had a CT colonography?

☐ Yes ☐ No ☐ Don't Know

If yes,

1. How many times have you had a CT colonography? ☐☐ times
2. How long ago was your last CT colonography? ☐☐.☐☐ years

VII. Family History Screen

A. Have any of your biological grandparents ever been diagnosed with colon or rectal cancer?

☐ Yes ☐ No ☐ Don't Know

If yes,

1. How many? ☐☐

B. Have any of your biological aunts or uncles ever been diagnosed with colon or rectal cancer?

☐ Yes ☐ No ☐ Don't Know

If yes,

1. How many? ☐☐

© 2023 Robertson DJ et al. *JAMA Network Open.*

Site No. ☐☐ **ID No.** ☐☐☐☐☐ **Date Form Completed (mm/dd/yyyy)** ☐☐ / ☐☐ / ☐☐☐☐

C. Have any of your biological nieces or nephews **EVER** been diagnosed with colon or rectal cancer?

☐ Yes ☐ No ☐ Don't Know

If yes,

1. How many? ☐☐

VIII. Height and Weight

A. Height

☐☐ inches

B. Weight

☐☐☐ pounds

IX. MILITARY SERVICE HISTORY

A. Have you ever served in the U.S. military?

☐ Active Duty ☐ Reserves Only ☐ Refused to Answer **(Skip to Section X)**

B. When did you serve ? **(check all that apply)**

☐ Prior to World War II (Nov 1941 or earlier)
☐ World War II (Dec 1941-Dec 1946)
☐ Jan 1947-June 1950
☐ Korean Conflict (July 1950-Jan 1955)
☐ Feb 1955-July 1964
☐ Vietnam Conflict (Aug 1964-April 1975)
☐ May 1975-July 1990
☐ Persian Gulf War (Aug 1990-Feb 1991)
☐ March 1991-Sept 2001
☐ Afghanistan/Iraq Conflict (Oct 2001- Present)
☐ Other war/conflict: ☐☐☐☐☐☐☐☐☐☐☐☐☐☐☐☐☐☐

© 2023 Robertson DJ et al. *JAMA Network Open.*

Site No. ☐☐ **ID No.** ☐☐☐☐☐ **Date Form Completed (mm/dd/yyyy)** ☐☐ / ☐☐ / ☐☐☐☐

C. Did you serve outside the United States?

☐ Yes ☐ No

D. What branch of service ? **(check all that apply)**

☐ Army ☐ Navy ☐ Marine Corps
☐ Coast Guard ☐ Air Force ☐ National Guard
☐ Merchant Marine ☐ NOAA ☐ Public Health Service

X. MEDICATIONS

A. VITAMINS

1. Do you currently take a multivitamin at least once a week on a regular basis?

   ☐ Yes ☐ No ☐ Don't Know

2. Do you currently take a calcium supplement at least once a week on a regular basis (Tums, Viactiv, etc.)?

   ☐ Yes ☐ No ☐ Don't Know

3. Do you currently take a Vitamin D supplement at least once a week on a regular basis?

   ☐ Yes ☐ No ☐ Don't Know

B. ASPIRIN-TYPE MEDICATIONS **(show med list to participant)**

1. Do you currently take aspirin-type medications?

   ☐ Yes ☐ No **(go to Question B.2)** ☐ Don't Know **(go to Question B.2)**

   a. How often do you take aspirin-type medications?

   Times ☐☐☐

   (check one)
   ○ Per day
   ○ Per week
   ○ Per month

© 2023 Robertson DJ et al. *JAMA Network Open.*

Site No. ☐☐ **ID No.** ☐☐☐☐☐ **Date Form Completed (mm/dd/yyyy)** ☐☐ / ☐☐ / ☐☐☐☐

b. How long have you taken aspirin-type medications?

☐☐ years and ☐☐ months

c. How many pills do you take each time?

☐☐ pills

d. What strengths do you usually take?

☐ "Baby" or "Adult Low-Dose" aspirin (81 mg)
☐ Half of one "Regular Strength" pill (approximately 162 mg)
☐ "Regular Strength" or normal dosage (325 mg)
☐ "Extra Strength" (500 mg or above)
☐ Don't Know

SKIP to Section C.

B.2 In the **past**, have you taken aspirin-type medications?

☐ Yes ☐ No **(go to Section C)** ☐ Don't Know **(go to Section C)**

If yes,

a. How often did you take aspirin-type medications?

Times ☐☐☐

(check one)
○ Per day
○ Per week
○ Per month

b. How long did you take aspirin-type medication?

☐☐ years and ☐☐ months

© 2023 Robertson DJ et al. *JAMA Network Open.*

Site No. ☐☐ **ID No.** ☐☐☐☐☐ **Date Form Completed (mm/dd/yyyy)** ☐☐ / ☐☐ / ☐☐☐☐

c. How many pills did you take each time?

☐☐ pills

d. What strength did you usually take? **(check one)**

☐ "Baby" or "Adult Low-Dose" aspirin (81 mg)

☐ Half of one "Regular Strength" pill (approximately 162 mg)

☐ "Regular Strength" or normal dosage (325 mg)

☐ "Extra Strength" (500 mg or above)

☐ Don't Know

C. IBUPROFEN-TYPE MEDICATIONS **(show med list to participant)**

1. Do you currently take Non Steroidal Anti-Inflammatory Drugs (NSAIDS) like Ibuprofen or Advil?

☐ Yes ☐ No **(go to Question C.2)** ☐ Don't Know **(go to Question C.2)**

a. How often do you take NSAIDS?

Times ☐☐☐ **(check one)**
○ Per day
○ Per week
○ Per month

b. How long have you taken NSAIDS?

☐☐ years and ☐☐ months

c. How many pills do you take each time?

☐☐ pills

© 2023 Robertson DJ et al. *JAMA Network Open.*

Site No. ☐☐ **ID No.** ☐☐☐☐☐ **Date Form Completed (mm/dd/yyyy)** ☐☐ / ☐☐ / ☐☐☐☐

d. What strength pills do you usually take?

☐ Over the counter (Regular Strength)
☐ Prescription Strength (Extra Strength)

Skip to Section D.

C.2 In the **past,** have you taken Non Steroidal Anti-Inflammatory Drugs (NSAIDS), like Ibuprofen or Advil?

☐ Yes ☐ No **(go to Section D)** ☐ Don't Know **(go to Section D)**

If yes,

a. How often did you take NSAIDS ?

Times ☐☐☐ **(check one)**
○ Per day
○ Per week
○ Per month

b. How long have you taken NSAIDS?

☐☐ years and ☐☐ months

c. How many pills did you usually take each time?

☐☐ pills

d. What strength pills did you usually take? **(check one)**

☐ Over the counter (Regular Strength)
☐ Prescription Strength (Extra Strength)

© 2023 Robertson DJ et al. *JAMA Network Open.*

Site No. ☐☐ **ID No.** ☐☐☐☐☐ **Date Form Completed (mm/dd/yyyy)** ☐☐ / ☐☐ / ☐☐☐☐

D. COXIB MEDICATIONS

1. Have you ever taken Vioxx (rofecoxib), Celebrex (celecoxib), or Bextra (valdecoxib) at least once a week?

   ☐ Yes ☐ No **(go to Section E)** ☐ Don't Know **(go to Section E)**

   If yes,

   a. Are you currently taking a coxib medication?

      ☐ Yes ☐ No ☐ Don't Know

E. STATIN TYPE MEDICATIONS **(show med list to participant)**

1. Do you currently take a statin cholesterol-lowering medication?

   ☐ Yes ☐ No **(go to Question E.2)** ☐ Don't Know **(go to Question E.2)**

   If yes,

   a. How often do you take this cholesterol-lowering medication?

      Times ☐☐

      (check one)
      ○ Per day
      ○ Per week
      ○ Per month

   b. How long have you taken a cholesterol-lowering medication?

      ☐☐ years and ☐☐ months

   SKIP to Section XI.

© 2023 Robertson DJ et al. *JAMA Network Open.*

Site No. ☐☐ **ID No.** ☐☐☐☐☐ **Date Form Completed (mm/dd/yyyy)** ☐☐ / ☐☐ / ☐☐☐☐

E 2. In the **past**, have you taken a statin cholesterol-lowering medications?

☐ Yes ☐ No **(go to Section XI)** ☐ Don't Know **(go to Section XI)**

If yes,

a. How often did you take this cholesterol-lowering medication?

Times ☐☐ **(check one)**
○ Per day
○ Per week
○ Per month

b. How long did you take a cholesterol-lowering medication?

☐☐ years and ☐☐ months

XI. Baseline Healthcare Utilization

A. Travel

1. How far do you travel one way to the VA facility where you usually receive care?

☐☐☐ miles

2. How long does it take you to travel one way to the VA facility where you usually receive care?

☐☐☐ minutes

3. How do you usually travel to the VA facility where you usually receive care? **(check one)**

☐ Drive myself
☐ Others (friends/family) drive me
☐ Public transportation (including bus, train or taxi)
☐ Disabled American Veterans (DAV) van or shuttle
☐ Other:specify ☐☐☐☐☐☐☐☐☐☐☐☐

© 2023 Robertson DJ et al. *JAMA Network Open.*

Site No.	ID No.	Date Form Completed (mm/dd/yyyy)
☐☐	☐☐☐☐☐	☐☐ / ☐☐ / ☐☐☐☐

B. Health Insurance Coverage:

1. Are you billed for a co-pay when you receive VA care?

☐ Yes ☐ No

2. In the past year, were you covered by health insurance in addition to Veterans health benefits?

☐ Yes ☐ No

a. If yes, please indicate which insurance provided you with coverage?

(Please mark all that apply)

☐ Private Health Care Insurance
☐ Medicare
☐ Medicaid
☐ Tricare
☐ Other

b. Frequency of Non-VA health care use:

1. When you need medical care, how often do you use **non** -VA health care providers?

☐ Frequently (>75% of the time)
☐ About half of the time
☐ Rarely (<25% of the time)
☐ Never

© 2023 Robertson DJ et al. *JAMA Network Open.*

Site No. ☐☐ ID No. ☐☐☐☐☐ Date Form Completed (mm/dd/yyyy) ☐☐/☐☐/☐☐☐☐

XII. **Physical Activity Questionnaire**

Read: I am going to ask you about the time you spent being physically active in the last 7 days. Please answer each question even if you do not consider yourself an active person. Think about the activities you do: at work, as part of your house and yard work, to get from place to place, and in your spare time for recreation, exercise or sport.

A. Vigorous Physical Activity during the last 7 days.

Read: Now,think about all the vigorous activities which take *hard physical effort* that you did in the last 7 days. Vigorous activities make you breathe much harder than normal and may include heavy lifting,digging, aerobics,or fast bicycling. Think only about those physical activities that you did for at least 10 minutes at a time.

1. During the last **7 days**, on how many days did you do vigorous physical activities?

☐ Days ☐ Don't Know/Not Sure ☐ Refused to answer

2. How much time did you usually spend doing **vigorous** physical activities on one of those days?

☐☐ Hours ☐☐ Minutes ☐ Don't Know/Not Sure ☐ Refused to answer

B. Moderate Physical Activity during the past 7 days.

Read: Now think about the activities which take *moderate physical effort* that you did in the last 7 days. Moderate physical activities make you breathe somewhat harder than normal and may include carrying light loads, bicycling at a regular pace, or doubles tennis. Do not include walking. Again,think about only those physical activities that you did for at least 10 minutes at a time.

1. During the last **7 days**, on how many days did you do **moderate** physical activities?

☐ Days ☐ Don't Know/Not Sure ☐ Refused to answer

© 2023 Robertson DJ et al. *JAMA Network Open.*

Site No. ☐☐ ID No. ☐☐☐☐☐ Date Form Completed (mm/dd/yyyy) ☐☐ / ☐☐ / ☐☐☐☐

2. How much time did you usually spend doing **moderate** physical activities on one of those days?

☐☐ Hours ☐☐ Minutes ☐ Don't Know/Not Sure ☐ Refused to answer

C. Time spent walking in the past 7 days.

Read: Now think about the time you spent walking in the last 7 days. This includes at work and at home, walking to travel from place to place, and any other walking that you might do solely for recreation, sport exercise or leisure.

1. During the last **7 days**, on how many days did you **walk** for at least 10 minutes at a time?

☐ Days ☐ Don't Know/Not Sure ☐ Refused to answer

2. How much time did you usually spend **walking** on one of those days?

☐☐ Hours ☐☐ Minutes ☐ Don't Know/Not Sure ☐ Refused to answer

D. Time spent sitting in the past 7 days.

Read: Now think about the time you spent sitting in the last 7 days. This includes at work and at home, while doing course work, and during leisure time. This might include time spent sitting at a desk, visiting friends, reading or sitting or lying down to watch television.

1. During the last 7 days, how much time did you usually spend **sitting** on a **week day**?

☐☐ Hours ☐☐ Minutes ☐ Don't Know/Not Sure ☐ Refused to answer

© 2023 Robertson DJ et al. *JAMA Network Open.*

Site No. ☐☐ **ID No.** ☐☐☐☐☐ **Date Form Completed (mm/dd/yyyy)** ☐☐ / ☐☐ / ☐☐☐☐

XIII. TOBACCO USE

A. Have you smoked at least 100 cigarettes in your lifetime?

☐ Yes ☐ No **(go to Section XIV)** ☐ **Refused (go to section XIV)**

B. Do you currently smoke cigarettes?

☐ Yes ☐ No **(go to Question D)** ☐ **Refused (go to Question D)**

C. How many cigarettes do you usually smoke **in a week**?

☐☐☐

D. How old were you when you first started smoking cigarettes on a regular basis?

☐☐ years old

E. Some people stop smoking and start again one or more times. In total, how many years in your lifetime have you smoked cigarettes on a regular basis?

☐☐ years

F. If you no longer smoke cigarettes, how old were you when you stopped smoking cigarettes?

☐☐ years old

G. If you no longer smoke cigarettes, on average, how many cigarettes did you smoke **in a week** before you quit?

☐☐☐

© 2023 Robertson DJ et al. *JAMA Network Open.*

VA CSP #577
Colonoscopy vs.Fecal Immunochemical Test in Reducing Mortality from Colorectal Cancer
Form 02 - Baseline

Site No. ☐☐ ID No. ☐☐☐☐☐ Date Form Completed (mm/dd/yyyy) ☐☐ / ☐☐ / ☐☐☐☐

XIV. ALCOHOL USE

A. The following questions ask about the types and amounts of alcoholic beverages you drank in the **last year**.

1. Did you drink beer? ☐ Yes ☐ No **(go to Question 2)** ☐ Refused **(go to Question 2)**

   a. If yes, how often did you drink beer? ☐☐☐ No. of Days
   ☐ Per Week
   ☐ Per Month
   ☐ Per Year
   ☐ Refused to Answer

   b. On those days that you drank beer, on average, how many beers did you have? ☐☐ (No. of beers)
   ☐ Refused to Answer ☐ Don't Know

2. Did you drink wine ? ☐ Yes ☐ No **(go to Question 3)** ☐ Refused **(go to Question 3)**

   a. If yes, how often did you drink wine? ☐☐☐ No. of Days
   ☐ Per Week
   ☐ Per Month
   ☐ Per Year
   ☐ Refused to Answer

   b. On those days that you drank wine, on average, how many glasses did you have? ☐☐ (No. glasses of wine)
   ☐ Refused to Answer ☐ Don't Know

© 2023 Robertson DJ et al. *JAMA Network Open.*

VA CSP #577
Colonoscopy vs.Fecal Immunochemical Test in Reducing Mortality from Colorectal Cancer
Form 02 - Baseline

Site No. ☐☐ **ID No.** ☐☐☐☐☐ **Date Form Completed (mm/dd/yyyy)** ☐☐ / ☐☐ / ☐☐☐☐

3. Did you drink hard liquor? ☐ Yes ☐ No **(go to Section B)** ☐ Refused **(go to Section B)**

a. If yes, how often did you drink hard liquor? ☐☐☐ No. of Days
☐ Per Week
☐ Per Month
☐ Per Year
☐ Refused to Answer

b. On those days that you drank hard liquor, on average, how many shots did you have? ☐☐ (No. of Shots)

☐ Refused to Answer ☐ Don't Know

B.Considering all types of alcoholic beverages, during the past 30 days, did you have **(5 for Males,4 for Females)** or more drinks on a single occasion?

☐ Yes ☐ No ☐ Don't Know/Not Sure ☐ Refused to Answer

If yes, ☐☐ number of times.

Survey completed for Males.

Please complete Baseline Form 3 for Females.

Staff Initials ☐☐☐

© 2023 Robertson DJ et al. *JAMA Network Open.*

6267194285

VA CSP #577
Colonoscopy vs. Fecal Immunochemical Test in Reducing Mortality from Colorectal Cancer (CONFIRM)
Form 03 Baseline - Females Only

Site No.	ID No.	Date Form Completed (mm/dd/yyyy)
☐☐	☐☐☐☐☐	☐☐ / ☐☐ / ☐☐☐☐

I. GYNECOLOGICAL AND HORMONE-RELATED HISTORY

A. Do you still have menstrual periods?

☐ Yes ☐ No ☐ Don't Know/Declined to answer

If no,

1. How old were you when your periods stopped permanently (for at least 12 months)?

☐☐ years old

B. Have you **EVER** used birth control pills or other hormonal contraceptives (implants or injections) for at least one year?

☐ Yes ☐ No ☐ Don't Know/Declined to answer

If yes,

1. Are you currently using birth control pills or other hormonal contraceptives?

☐ Yes ☐ No ☐ Don't Know/Declined to answer

2. How long have you used birth control pills or other hormonal contraceptives?

☐☐ years AND ☐☐ months

C. Have you **EVER** taken hormone replacement therapy for menopause for at least 6 months?

☐ Yes ☐ No ☐ Don't Know/Declined to answer

If yes,

1. Are you currently taking hormone replacement therapy for menopause?

☐ Yes ☐ No ☐ Don't Know/Declined to answer

2. How long have you used hormone replacement therapy for menopause?

☐☐ years AND ☐☐ months

© 2023 Robertson DJ et al. *JAMA Network Open.*

VA CSP #577
Colonoscopy vs. Fecal Immunochemical Test in Reducing Mortality from Colorectal Cancer (CONFIRM)
Form 03 Baseline - Females Only

Site No.	ID No.	Date Form Completed (mm/dd/yyyy)
☐☐	☐☐☐☐☐	☐☐ / ☐☐ / ☐☐☐☐

D. Have you **EVER** taken an estrogen-only pill or patch (such as Premarin) as hormone replacement therapy for menopause or other reasons?

☐ Yes ☐ No ☐ Don't Know/Declined to answer

If yes,

1. Are you currently taking an estrogen-only pill or patch (such as Premarin) as hormone replacement therapy for menopause or other reasons?

   ☐ Yes ☐ No ☐ Don't Know/Declined to answer

2. How long have you used an estrogen-only pill or patch?

   ☐☐ years AND ☐☐ months

E. Have you **EVER** taken progesterone or progestin along with estrogens for menopause or for other reasons? Progesterone or progestin is frequently prescribed by doctors along with estrogen as hormone replacement therapy for menopause. Some common brands are Provera and Prem-Pro.

☐ Yes ☐ No ☐ Don't Know/Declined to answer

If yes,

1. Are you currently taking progesterone or progestin along with estrogens for menopause or for other reasons?

   ☐ Yes ☐ No ☐ Don't Know/Declined to answer

2. How long have you used progesterone or progestin along with estrogens?

   ☐☐ years AND ☐☐ months

F. Have you **EVER** taken progesterone or progestin alone (without other prescribed hormones such as estrogen) as hormone replacement therapy for menopause or for other reasons?

☐ Yes ☐ No ☐ Don't Know/Declined to answer

If yes,

1. Are you currently taking progesterone or progestin alone as hormone replacement therapy for menopause or for other reasons?

   ☐ Yes ☐ No ☐ Don't Know/Declined to answer

2. How long have you used progesterone or progestin alone as hormone replacement therapy for menopause or for other reasons?

   ☐☐ years AND ☐☐ months

© 2023 Robertson DJ et al. *JAMA Network Open.*

VA CSP #577
Colonoscopy vs. Fecal Immunochemical Test in Reducing Mortality from Colorectal Cancer (CONFIRM)
Form 03 Baseline - Females Only

Site No.	ID No.	Date Form Completed (mm/dd/yyyy)
☐☐	☐☐☐☐☐	☐☐ / ☐☐ / ☐☐☐☐

G. Have you **EVER** taken a combination of estrogen and testosterone for menopause or for other reasons?

☐ Yes ☐ No ☐ Don't Know/Declined to answer

If yes,

1. Are you currently taking a combination of estrogen and testosterone for menopause or for other reasons?

   ☐ Yes ☐ No ☐ Don't Know/Declined to answer

2. How long have you used a combination of estrogen and testosterone for menopause or for other reasons?

   ☐☐ years AND ☐☐ months

H. Have you **EVER** taken Tamoxifen or Raloxifene (Evista)?

☐ Yes ☐ No ☐ Don't Know/Declined to answer

If yes,

1. Are you currently taking Tamoxifen or Raloxifene (Evista)?

   ☐ Yes ☐ No ☐ Don't Know/Declined to answer

2. How long have you used Tamoxifen or Raloxifene (Evista)?

   ☐☐ years AND ☐☐ months

Staff Initials ☐☐☐

© 2023 Robertson DJ et al. *JAMA Network Open.*
