# Supplementary material for: Baseline Features and Reasons for Nonparticipation in the Colonoscopy Versus Fecal Immunochemical Test in Reducing Mortality From Colorectal Cancer (CONFIRM) Study, a Colorectal Cancer Screening Trial
Source: JAMA Netw Open. 2023 Jul 11;6(7):e2321730. doi: 10.1001/jamanetworkopen.2023.21730 (PMC10336619; doi:10.1001/jamanetworkopen.2023.21730)
Supplement: Supplement 2. — Nonauthor Collaborators [file jamanetwopen-e2321730-s002.pdf]

*Group Name(s): CONFIRM Study Group							
***First Name and Middle Initial(s)**	***Last Name**	***Suffix (eg, Jr, III)**	Academic Degrees	Institution	Location (city, state/province, country)	Role or Contribution, eg, chair, principal investigator	Group (if more than 1 Group listed in the byline) and/or Subgroup (eg, Steering Committee)
Elaine	Kleiner		PhD	West Haven Cooperative Studies Program Coordinating Center (WH-CSPCC)	West Haven	Data Analyst	WH-CSP
Kathy	Sullivan		MS	West Haven Cooperative Studies Program Coordinating Center (WH-CSPCC)	West Haven	Data Analyst	WH-CSP
John	O'Leary			West Haven Cooperative Studies Program Coordinating Center (WH-CSPCC)	West Haven	Data Analyst	WH-CSP
Wai (Susannah)	Chiu		MS	West Haven Cooperative Studies Program Coordinating Center (WH-CSPCC)	West Haven	Data Analyst	WH-CSP
Hairong	Huang			West Haven Cooperative Studies Program Coordinating Center (WH-CSPCC)	West Haven	Data Analyst	WH-CSP
John	Russo			West Haven Cooperative Studies Program Coordinating Center (WH-CSPCC)	West Haven	Data Analyst	WH-CSP
Gary	Johnson		MS	West Haven Cooperative Studies Program Coordinating Center (WH-CSPCC)	West Haven	Acting Deputy Center Director	WH-CSP
Peg	Antonelli			West Haven Cooperative Studies Program Coordinating Center (WH-CSPCC)	West Haven	Associate Center Director for Administrative Operations	WH-CSP
Amy	Burns		JD	West Haven Cooperative Studies Program Coordinating Center (WH-CSPCC)	West Haven	Associate Director for Quality Assurance	WH-CSP
Peter	Peduzzi		PhD	West Haven Cooperative Studies Program Coordinating Center (WH-CSPCC)	West Haven	Center Director	WH-CSP

*First name, last name, and suffix (if applicable) are required and will appear in PubMed.

***First Name and Middle Initial(s)**	***Last Name**	***Suffix (eg, Jr, III)**	Academic Degrees	Institution	Location (city, state/province, country)	Role or Contribution, eg, chair, principal investigator	Group (if more than 1 Group listed in the byline) and/or Subgroup (eg, Steering Committee)
Maria	Ciarleglio		PhD	West Haven Cooperative Studies Program Coordinating Center (WH-CSPCC)	West Haven	Biostatistician	WH-CSP
Theresa	O'Connor		MPH, PhD	West Haven Cooperative Studies Program Coordinating Center (WH-CSPCC)	West Haven	Biostatistician	WH-CSP
Robert	Wallace		ScD	West Haven Cooperative Studies Program Coordinating Center (WH-CSPCC)	West Haven	Biostatistician	WH-CSP
Alicia	Williams		MS	West Haven Cooperative Studies Program Coordinating Center (WH-CSPCC)	West Haven	Biostatistician	WH-CSP
Lynn	Tommessilli		BS	West Haven Cooperative Studies Program Coordinating Center (WH-CSPCC)	West Haven	Project Manager	WH-CSP
Sakasha	Taylor		MS	West Haven Cooperative Studies Program Coordinating Center (WH-CSPCC)	West Haven	Project Manager	WH-CSP
Sarah	Pitts		MS	West Haven Cooperative Studies Program Coordinating Center (WH-CSPCC)	West Haven	Project Manager	WH-CSP
Lesley W.	Mancini			West Haven Cooperative Studies Program Coordinating Center (WH-CSPCC)	West Haven	Project Manager	WH-CSP
Peggy	O'Brien		BA	West Haven Cooperative Studies Program Coordinating Center (WH-CSPCC)	West Haven	Research Associate	WH-CSP
Susan	O'Neil		MHA	West Haven Cooperative Studies Program Coordinating Center (WH-CSPCC)	West Haven	Research Associate	WH-CSP
Mavis	Perry			West Haven Cooperative Studies Program Coordinating Center (WH-CSPCC)	West Haven	Research Associate	WH-CSP

*First name, last name, and suffix (if applicable) are required and will appear in PubMed.

***First Name and Middle Initial(s)**	***Last Name**	***Suffix (eg, Jr, III)**	Academic Degrees	Institution	Location (city, state/province, country)	Role or Contribution, eg, chair, principal investigator	Group (if more than 1 Group listed in the byline) and/or Subgroup (eg, Steering Committee)
Vanessa	McBride		BS	West Haven Cooperative Studies Program Coordinating Center (WH-CSPCC)	West Haven	Research Associate	WH-CSP
Wanda	Carr			West Haven Cooperative Studies Program Coordinating Center (WH-CSPCC)	West Haven	Research Associate	WH-CSP
Carmelene	Joncas		RN	West Haven Cooperative Studies Program Coordinating Center (WH-CSPCC)	West Haven	Quality Nurse	WH-CSP
Elizabeth	O'Donnell		RN	West Haven Cooperative Studies Program Coordinating Center (WH-CSPCC)			
Mike	Sather			CSP Clinical Research Pharmacy Coordinating Center (CSPCRPCC)	Albuquerque	CSPCRPCC Director	Office of Center's Director
Stuart	Warren			CSP Clinical Research Pharmacy Coordinating Center (CSPCRPCC)	Albuquerque	CSPCRPCC Director	Office of Center's Director
Todd	Conner			CSP Clinical Research Pharmacy Coordinating Center (CSPCRPCC)	Albuquerque	CSPCRPCC Director	Office of Center's Director
Ernestine	Honstein			CSP Clinical Research Pharmacy Coordinating Center (CSPCRPCC)	Albuquerque	Research Assistant	Administrative Section
Theresa	Sutton			CSP Clinical Research Pharmacy Coordinating Center (CSPCRPCC)	Albuquerque	Research Assistant	Administrative Section
Tracey	Putnam			CSP Clinical Research Pharmacy Coordinating Center (CSPCRPCC)	Albuquerque	Research Assistant	Administrative Section

*First name, last name, and suffix (if applicable) are required and will appear in PubMed.

***First Name and Middle Initial(s)**	***Last Name**	***Suffix (eg, Jr, III)**	Academic Degrees	Institution	Location (city, state/province, country)	Role or Contribution, eg, chair, principal investigator	Group (if more than 1 Group listed in the byline) and/or Subgroup (eg, Steering Committee)
Kevin	Branham			CSP Clinical Research Pharmacy Coordinating Center (CSPCRPCC)	Albuquerque	Chief Administrative Section	Administrative Section
Dean	Argyres			CSP Clinical Research Pharmacy Coordinating Center (CSPCRPCC)	Albuquerque	Chief Biopharmaceutics Laboratory Section	Biopharmaceutics Laboratory Section
Dianne	Peterson			CSP Clinical Research Pharmacy Coordinating Center (CSPCRPCC)	Albuquerque	Chemist	Biopharmaceutics Laboratory Section
April	Kennedy			CSP Clinical Research Pharmacy Coordinating Center (CSPCRPCC)	Albuquerque	Chemist	Biopharmaceutics Laboratory Section
Toni	Carrick			CSP Clinical Research Pharmacy Coordinating Center (CSPCRPCC)	Albuquerque	Chemist	Biopharmaceutics Laboratory Section
Carlos	Apodaca			CSP Clinical Research Pharmacy Coordinating Center (CSPCRPCC)	Albuquerque	Technition	Biopharmaceutics Laboratory Section
Gary	Eden			CSP Clinical Research Pharmacy Coordinating Center (CSPCRPCC)	Albuquerque	Chemist	Biopharmaceutics Laboratory Section
Talaya	Martinez			CSP Clinical Research Pharmacy Coordinating Center (CSPCRPCC)	Albuquerque	Production Team Coordinator	Clinical Materials Management Section
Cindy	Tripp			CSP Clinical Research Pharmacy Coordinating Center (CSPCRPCC)	Albuquerque	Production Team Coordinator	Clinical Materials Management Section
Michelle	Nicholson			CSP Clinical Research Pharmacy Coordinating Center (CSPCRPCC)	Albuquerque	Production Team Coordinator	Clinical Materials Management Section

Supplemental Online Content: Nonauthor Collaborators
*First name, last name, and suffix (if applicable) are required and will appear in PubMed.

***First Name and Middle Initial(s)**	***Last Name**	***Suffix (eg, Jr, III)**	Academic Degrees	Institution	Location (city, state/province, country)	Role or Contribution, eg, chair, principal investigator	Group (if more than 1 Group listed in the byline) and/or Subgroup (eg, Steering Committee)
Amy	Schwartz			CSP Clinical Research Pharmacy Coordinating Center (CSPCRPCC)	Albuquerque	Production Team Coordinator	Clinical Materials Management Section
Monique	Felix			CSP Clinical Research Pharmacy Coordinating Center (CSPCRPCC)	Albuquerque	Production Team Coordinator	Clinical Materials Management Section
Angela	Ward			CSP Clinical Research Pharmacy Coordinating Center (CSPCRPCC)	Albuquerque	Clinical Label Specialist	Information Systems Section
Leanne	Marquez			CSP Clinical Research Pharmacy Coordinating Center (CSPCRPCC)	Albuquerque	Computer Assistant	Information Systems Section
Karsondra	Lovato			CSP Clinical Research Pharmacy Coordinating Center (CSPCRPCC)	Albuquerque	Computer Assistant	Information Systems Section
Neil	Cochran			CSP Clinical Research Pharmacy Coordinating Center (CSPCRPCC)	Albuquerque	Computer Assistant	Information Systems Section
Steve	Tapia			CSP Clinical Research Pharmacy Coordinating Center (CSPCRPCC)	Albuquerque	Computer Assistant	Information Systems Section
Melissa	VanRaden			CSP Clinical Research Pharmacy Coordinating Center (CSPCRPCC)	Albuquerque	Software Developer	Information Systems Section
Carol	Badgett			CSP Clinical Research Pharmacy Coordinating Center (CSPCRPCC)	Albuquerque	Database Administrator	Information Systems Section
Zach	Taylor			CSP Clinical Research Pharmacy Coordinating Center (CSPCRPCC)	Albuquerque	Software Developer	Information Systems Section
Jimmy	Pontzer			CSP Clinical Research Pharmacy Coordinating Center (CSPCRPCC)	Albuquerque	Software Developer	Information Systems Section

*First name, last name, and suffix (if applicable) are required and will appear in PubMed.

***First Name and Middle Initial(s)**	***Last Name**	***Suffix (eg, Jr, III)**	Academic Degrees	Institution	Location (city, state/province, country)	Role or Contribution, eg, chair, principal investigator	Group (if more than 1 Group listed in the byline) and/or Subgroup (eg, Steering Committee)
Bert	Dorman			CSP Clinical Research Pharmacy Coordinating Center (CSPCRPCC)	Albuquerque	Quality Manager	Quality Control
Michael	Chavez			CSP Clinical Research Pharmacy Coordinating Center (CSPCRPCC)	Albuquerque	Quality Manager	Quality Control
Sharon	Georg			CSP Clinical Research Pharmacy Coordinating Center (CSPCRPCC)	Albuquerque	Quality Manager	Quality Control
Terri	Templton			CSP Clinical Research Pharmacy Coordinating Center (CSPCRPCC)	Albuquerque	Quality Manager	Quality Control
Lawrence	Calais			CSP Clinical Research Pharmacy Coordinating Center (CSPCRPCC)	Albuquerque	Director, SMART	Site Monitoring Auditing and Resource Team (SMART)
Claire	Haakensen			CSP Clinical Research Pharmacy Coordinating Center (CSPCRPCC)	Albuquerque	Chief SMART	Site Monitoring Auditing and Resource Team (SMART)
Barbara	Curtis			CSP Clinical Research Pharmacy Coordinating Center (CSPCRPCC)	Albuquerque	SMART Program Manager	Site Monitoring Auditing and Resource Team (SMART)
Michelle	Prehoda			CSP Clinical Research Pharmacy Coordinating Center (CSPCRPCC)	Albuquerque	SMART Program Manager	Site Monitoring Auditing and Resource Team (SMART)
Cindy	Colling			CSP Clinical Research Pharmacy Coordinating Center (CSPCRPCC)	Albuquerque	AE/SAE Specialist	RACC

*First name, last name, and suffix (if applicable) are required and will appear in PubMed.

***First Name and Middle Initial(s)**	***Last Name**	***Suffix (eg, Jr, III)**	Academic Degrees	Institution	Location (city, state/province, country)	Role or Contribution, eg, chair, principal investigator	Group (if more than 1 Group listed in the byline) and/or Subgroup (eg, Steering Committee)
Dave	Older			CSP Clinical Research Pharmacy Coordinating Center (CSPCRPCC)	Albuquerque	Contracting Officer's Representative (COR)	Finance Section
Gloria	Apodaca			CSP Clinical Research Pharmacy Coordinating Center (CSPCRPCC)	Albuquerque	Budget Analyst	Finance Section
Julia	Sova			CSP Clinical Research Pharmacy Coordinating Center (CSPCRPCC)	Albuquerque	Budget Technician	Finance Section
Mike	Sather			CSP Clinical Research Pharmacy Coordinating Center (CSPCRPCC)	Albuquerque	CSPCRPCC Director	Office of Center's Director
Stuart	Warren			CSP Clinical Research Pharmacy Coordinating Center (CSPCRPCC)	Albuquerque	CSPCRPCC Director	Office of Center's Director
Todd	Conner			CSP Clinical Research Pharmacy Coordinating Center (CSPCRPCC)	Albuquerque	CSPCRPCC Director	Office of Center's Director
Magnolia C.	Klee		BS	West Coast Co-Chair Office,VA Puget Sound Health Care System	Seattle	National Research Assistant	
Antigone	Wolfram-Aduan		PhD	West Coast Co-Chair Office,VA Puget Sound Health Care System	Seattle	National Research Assistant	
Michael J.	Ulatowski		MPH	West Coast Co-Chair Office, VA Puget Sound Health Care System	Seattle	National Research Assistant	
Brittney L.	Hamilton		MA	West Coast Co-Chair Office, VA Puget Sound Health Care System	Seattle	National Research Assistant	

*First name, last name, and suffix (if applicable) are required and will appear in PubMed.

***First Name and Middle Initial(s)**	***Last Name**	***Suffix (eg, Jr, III)**	Academic Degrees	Institution	Location (city, state/province, country)	Role or Contribution, eg, chair, principal investigator	Group (if more than 1 Group listed in the byline) and/or Subgroup (eg, Steering Committee)
Kayla M.	Bopp (Formerly England)		MS	West Coast Co-Chair Office, VA Puget Sound Health Care System	Seattle	National Research Assistant	
Gashia M.	Ford		BS	West Coast Co-Chair Office, VA Puget Sound Health Care System	Seattle	National Research Assistant	
Ngaio E.	Lace		BS	West Coast Co-Chair Office, VA Puget Sound Health Care System	Seattle	National Research Assistant	
William A.	McDonnell		BS	West Coast Co-Chair Office, VA Puget Sound Health Care System	Seattle	National Research Assistant	
Gina C.	Piehl		BS	West Coast Co-Chair Office, VA Puget Sound Health Care System	Seattle	National Research Assistant	
Nazleen	Patel-Ejarque		MPH	West Coast Co-Chair Office, VA Puget Sound Health Care System	Seattle	National Research Assistant	
Maegan	Berry		BS	East Coast Co-Chair Office, White River Junction VA Medical Center	White River Junction	National Research Assistant	
Eric A.	Strubeck		MS	East Coast Co-Chair Office, White River Junction VA Medical Center	White River Junction	National Research Assistant	
Leigh	Chesnut			East Coast Co-Chair Office, White River Junction VA Medical Center	White River Junction	National Research Assistant	
Laurie J.	Burridge			East Coast Co-Chair Office, White River Junction VA Medical Center	White River Junction	National Research Assistant	
Jennifer R.	Bean		NMT, CCA	East Coast Co-Chair Office, White River Junction VA Medical Center	White River Junction	National Biorepository Coordinator	

*First name, last name, and suffix (if applicable) are required and will appear in PubMed.

***First Name and Middle Initial(s)**	***Last Name**	***Suffix (eg, Jr, III)**	Academic Degrees	Institution	Location (city, state/province, country)	Role or Contribution, eg, chair, principal investigator	Group (if more than 1 Group listed in the byline) and/or Subgroup (eg, Steering Committee)
Lynda M.	Kowal			East Coast Co-Chair Office, White River Junction VA Medical Center	White River Junction	National Research Assistant	
Amy V.	Voorhees (Formerly Rose)		MPA, MPH	East Coast Co-Chair Office, White River Junction VA Medical Center	White River Junction	National Study Coordinator	
Amy	Ridley		BA, MS	East Coast Co-Chair Office, White River Junction VA Medical Center	White River Junction	National Research Assistant	
Heike B.	Croteau			East Coast Co-Chair Office, White River Junction VA Medical Center	White River Junction	National Research Assistant	
Kate A.	Bessett		BS	East Coast Co-Chair Office, White River Junction VA Medical Center	White River Junction	National Study Coordinator	
Carol	Fletcher		PhD, RN	VA Ann Arbor Healthcare System	Ann Arbor	Local Study Coordinator	
Michelle	Barbaresso		MPH	VA Ann Arbor Healthcare System	Ann Arbor	Local Study Coordinator	
Alysia	Drummond		MPH, RD	VA Ann Arbor Healthcare System	Ann Arbor	Local Study Coordinator	
Karen	Belanger		MS, RD	VA Ann Arbor Healthcare System	Ann Arbor	Local Study Coordinator	
Erin	McRobert		MSW	VA Ann Arbor Healthcare System	Ann Arbor	Local Study Coordinator	
Aimee	Myers			VA Ann Arbor Healthcare System	Ann Arbor	Local Study Coordinator	
Erika	Trumble		MPH	VA Ann Arbor Healthcare System	Ann Arbor	Local Study Coordinator	
Patricia	Rose		M. Div	VA Ann Arbor Healthcare System	Ann Arbor	Local Study Coordinator	

Supplemental Online Content: Nonauthor Collaborators
*First name, last name, and suffix (if applicable) are required and will appear in PubMed.

***First Name and Middle Initial(s)**	***Last Name**	***Suffix (eg, Jr, III)**	Academic Degrees	Institution	Location (city, state/province, country)	Role or Contribution, eg, chair, principal investigator	Group (if more than 1 Group listed in the byline) and/or Subgroup (eg, Steering Committee)
Aimee	Myers		BS	VA Ann Arbor Healthcare System	Ann Arbor	Research Assistant	
Anita	Biswas		BSN	VA Ann Arbor Healthcare System	Ann Arbor	Research Assistant	
Eric	Enache			VA Ann Arbor Healthcare System	Ann Arbor	Research Assistant	
Matthew	Shankin		MSW	VA Ann Arbor Healthcare System	Ann Arbor	Research Assistant	
Danielle	Strunk			VA Ann Arbor Healthcare System	Ann Arbor	Research Assistant	
Andrew	Simpson		MD	Atlanta VA Medical Center	Atlanta	Sub-Investigator	
Melanie	Harrison		MD	Atlanta VA Medical Center	Atlanta	Sub-Investigator	
Meena	Prasad		MD	Atlanta VA Medical Center	Atlanta	Sub-Investigator	
Pauline	Robinson		BSN, RN	Atlanta VA Medical Center	Atlanta	Local Study Coordinator	
Alegandro	Flores			Atlanta VA Medical Center	Atlanta	Research Assistant	
Sara	Jananeh			Atlanta VA Medical Center	Atlanta	Research Assistant	
Sridhar	Tanukonda			Atlanta VA Medical Center	Atlanta	Research Assistant	
Ghazal	Ahmadi-Izadi			Atlanta VA Medical Center	Atlanta	Research Assistant	
Shahrzad	Madihi			Atlanta VA Medical Center	Atlanta	Research Assistant	
Jean-Pierre	Raufman		MD	Baltimore VAMC (VA Maryland HCS)	Baltimore	Sub-Investigator	
Deborah	Grady		RN, MS, CCRC	Baltimore VAMC (VA Maryland HCS)	Baltimore	Local Study Coordinator	

Supplemental Online Content: Nonauthor Collaborators
*First name, last name, and suffix (if applicable) are required and will appear in PubMed.

*First Name and Middle Initial(s)	*Last Name	*Suffix (eg, Jr, III)	Academic Degrees	Institution	Location (city, state/province, country)	Role or Contribution, eg, chair, principal investigator	Group (if more than 1 Group listed in the byline) and/or Subgroup (eg, Steering Committee)
Angela	Britton			Baltimore VAMC (VA Maryland HCS)	Baltimore	Local Study Coordinator	
Carly	Goldstein			Baltimore VAMC (VA Maryland HCS)	Baltimore	Local Study Coordinator	
Carly	Goldstein			Baltimore VAMC (VA Maryland HCS)	Baltimore	Research Assistant	
Michelle	Thomas			Baltimore VAMC (VA Maryland HCS)	Baltimore	Research Assistant	
Veronica	Lee		RN	VA Boston Health Care System	Boston	Local Study Coordinator	
Myrdell	Belizaire			VA Boston Health Care System	Boston	Research Assistant	
Caroline	Costa			VA Boston Health Care System	Boston	Research Assistant	
Samuel	Davis		RN	VA Boston Health Care System	Boston	Research Assistant	
Makaila	Decker		BS	VA Boston Health Care System	Boston	Research Assistant	
Ashley	Marrama		BS	VA Boston Health Care System	Boston	Research Assistant	
Dorothy	Gilroy-Fanaras		RN	VA Boston Health Care System	Boston	Research Assistant	
Jesse	Gass		MPH	VA Boston Health Care System	Boston	Research Assistant	
Scott	Reece			VA Boston Health Care System	Boston	Research Assistant	
Desiree	Tobin			VA Boston Health Care System	Boston	Research Assistant	
Robert	Carroll		MD	Jesse Brown VA Medical Center	Chicago	Sub-Investigator	

Supplemental Online Content: Nonauthor Collaborators
*First name, last name, and suffix (if applicable) are required and will appear in PubMed.

***First Name and Middle Initial(s)**	***Last Name**	***Suffix (eg, Jr, III)**	Academic Degrees	Institution	Location (city, state/province, country)	Role or Contribution, eg, chair, principal investigator	Group (if more than 1 Group listed in the byline) and/or Subgroup (eg, Steering Committee)
Maria	Ferreira		MD	Jesse Brown VA Medical Center	Chicago	Sub-Investigator	
Dawn	Irvin			Jesse Brown VA Medical Center	Chicago	Local Study Coordinator	
Victoria	Morken			Jesse Brown VA Medical Center	Chicago	Research Assistant	
Michael	Covelli		MD	Louis A. Johnson VA Medical Center	Clarksburg	Sub-Investigator	
Teodoro	Medina		MD	Louis A. Johnson VA Medical Center	Clarksburg	Sub-Investigator	
Frank	Schiebel		MD	Louis A. Johnson VA Medical Center	Clarksburg	Sub-Investigator	
Anna	Shreves		RN	Louis A. Johnson VA Medical Center	Clarksburg	Local Study Coordinator	
Donald	Shriver		RN	Louis A. Johnson VA Medical Center	Clarksburg	Local Study Coordinator	
Yvonne	Frederick		RN	Louis A. Johnson VA Medical Center	Clarksburg	Local Study Coordinator	
Candice	Postle (formerly Matheny)			Lois A Johnson VA medical Center	Clarksburg	Research Assistant	
Jessica	Shaw			Lois A Johnson VA medical Center	Clarksburg	Research Assistant	
Dorothy	Pastor		RN, BSN	Louis Stokes VA Medical Center	Cleveland	Local Study Coordinator	
Anna	Armstrong		RN, BSN	Louis Stokes VA Medical Center	Cleveland	Local Study Coordinator	
Vinay	Kumaran			Louis Stokes VA Medical Center	Cleveland	Research Assistant	

Supplemental Online Content: Nonauthor Collaborators
*First name, last name, and suffix (if applicable) are required and will appear in PubMed.

*First Name and Middle Initial(s)	*Last Name	*Suffix (eg, Jr, III)	Academic Degrees	Institution	Location (city, state/province, country)	Role or Contribution, eg, chair, principal investigator	Group (if more than 1 Group listed in the byline) and/or Subgroup (eg, Steering Committee)
April Jessica	Pinto		MD	Louis Stokes VA Medical Center	Cleveland	Research Assistant	
Vanessa	Marshall			Louis Stokes VA Medical Center	Cleveland	Research Assistant	
Stacye	McLarty		MSN, RN	VA North Texas Health Care System	Dallas	Local Study Coordinator	
James	Carlton			VA North Texas Health Care System	Dallas	Local Study Coordinator	
Kenneth	Murphy		RN	VA North Texas Health Care System	Dallas	Local Study Coordinator	
Kourtney	McLemore		BA	VA North Texas Health Care System	Dallas	Research Assistant	
Eric	Mortensen		MD, MSc	VA North Texas Health Care System	Dallas	Director	Network of Dedicated Enrollment Sites (NODES)
James	LePage		PhD	VA North Texas Health Care System	Dallas	Director	Network of Dedicated Enrollment Sites (NODES)
Cyenthia	Willis			VA North Texas Health Care Syste	Dallas	Manager	Network of Dedicated Enrollment Sites (NODES)
Kenneth	Berman		MD	VA Eastern Colorado Health Care System	Denver	Sub-Investigator	
Theresa	Dunn		MS	VA Eastern Colorado Health Care System	Denver	Local Study Coordinator	
Jeannine	Espinoza			VA Eastern Colorado Health Care System	Denver	Local Study Coordinator	
Eugenia (Jean)	Schleski		MA, LPC	VA Eastern Colorado Health Care System	Denver	Local Study Coordinator	

*First name, last name, and suffix (if applicable) are required and will appear in PubMed.

*First Name and Middle Initial(s)	*Last Name	*Suffix (eg, Jr, III)	Academic Degrees	Institution	Location (city, state/province, country)	Role or Contribution, eg, chair, principal investigator	Group (if more than 1 Group listed in the byline) and/or Subgroup (eg, Steering Committee)
Stephanie	Shrader		RN	VA Eastern Colorado Health Care System	Denver	Local Study Coordinator	
Kevin	Gropp			VA Eastern Colorado Health Care System	Denver	Research Assistant	
Ziad	Kanaan		MD	John D. Dingell VA Medical Center	Detroit	Sub-Investigator	
Lawrence	Miller		MD	John D. Dingell VA Medical Center	Detroit	Sub-Investigator	
Mohammad	Anees		MD	John D. Dingell VA Medical Center	Detroit	Sub-Investigator	
Stephanie	Judd		MD	John D. Dingell VA Medical Center	Detroit	Sub-Investigator	
Vicki	Berchou		RN	John D. Dingell VA Medical Center	Detroit	Local Study Coordinator	
Deann	Balogh			John D. Dingell VA Medical Center	Detroit	Local Study Coordinator	
Elizabeth	Jones			John D. Dingell VA Medical Cente	Detroit	Local Study Coordinator	
Kathleen 'Kelly'	Covert		RN	John D. Dingell VA Medical Cente	Detroit	Local Study Coordinator	
Yolanda	Davis			John D. Dingell VA Medical Cente	Detroit	Local Study Coordinator	
Cynthia	Marbury		RN	John D. Dingell VA Medical Cente	Detroit	Local Study Coordinator	
MaryAnn	Rambus			John D. Dingell VA Medical Cente	Detroit	Local Study Coordinator	
Erin	Olgren			John D. Dingell VA Medical Cente	Detroit	Local Study Coordinator	
Ziad	Gellad		MD, MPH	Durham VA Medical Center	Durham	Sub-Investigator	

Supplemental Online Content: Nonauthor Collaborators
*First name, last name, and suffix (if applicable) are required and will appear in PubMed.

***First Name and Middle Initial(s)**	***Last Name**	***Suffix (eg, Jr, III)**	Academic Degrees	Institution	Location (city, state/province, country)	Role or Contribution, eg, chair, principal investigator	Group (if more than 1 Group listed in the byline) and/or Subgroup (eg, Steering Committee)
Brian	Sullivan		MD, MHS	Durham VA Medical Center	Durham	Sub-Investigator	
Teresa	Day			Durham VA Medical Center	Durham	Research Assistant	
Sima	Teehan		MD	VA New Jersey HCS	East Orange	Sub-Investigator	
Laura	Peters		RN	VA Central California Health Care System	Fresno	Local Study Coordinator	
Raquel	Hernandez-Chavez		RN	VA Central California Health Care System	Fresno	Local Study Coordinator	
Eva	Murphy			VA Central California Health Care System	Fresno	Local Study Coordinator	
Eva	Murphy			VA Central California Health Care System	Fresno	Local Study Coordinator	
Judy (Christine)	Cherry		RN	VA Central California Health Care System	Fresno	Local Study Coordinator	
Edwin	Graham		RN	VA Central California Health Care System	Fresno	Local Study Coordinator	
Judith	Johnson		RN	VA Central California Health Care System	Fresno	Research Assistant	
William	Wooten			VA Central California Health Care System	Fresno	Research Assistant	
Jesus	Trujillo		AS	VA Central California Health Care System	Fresno	Research Assistant	
Eva	Murphy		RN	VA Central California Health Care System	Fresno	Research Assistant	
Maurice	Harris		BA	VA Central California Health Care System	Fresno	Research Assistant	
Mary	Alizadeh			North Florida/South Georgia Veterans Health System	Gainesville	Sub-Investigator	

*First name, last name, and suffix (if applicable) are required and will appear in PubMed.

*First Name and Middle Initial(s)	*Last Name	*Suffix (eg, Jr, III)	Academic Degrees	Institution	Location (city, state/province, country)	Role or Contribution, eg, chair, principal investigator	Group (if more than 1 Group listed in the byline) and/or Subgroup (eg, Steering Committee)
Margaret	Lo		MD, FACP	North Florida/South Georgia Veterans Health System	Gainesville	Sub-Investigator	
Shannaz	Sultan		MD, MHSc	North Florida/South Georgia Veterans Health System	Gainesville	Sub-Investigator	
Leslie	Brown		BA	North Florida/South Georgia Veterans Health System	Gainesville	Local Study Coordinator	
Lauren	Koster		BSN	North Florida/South Georgia Veterans Health System	Gainesville	Local Study Coordinator	
Jennifer	LeLaurin		MPH	North Florida/South Georgia Veterans Health System	Gainesville	Local Study Coordinator	
Anne	Irwin		RN	North Florida/South Georgia Veterans Health System	Gainesville	Local Study Coordinator	
Nicole	Kay			North Florida/South Georgia Veterans Health System	Gainesville	Research Assistant	
Sean	Mckillip			North Florida/South Georgia Veterans Health System	Gainesville	Research Assistant	
Mark	Lowe			North Florida/South Georgia Veterans Health System	Gainesville	Research Assistant	
Seyhun	Yeralan			North Florida/South Georgia Veterans Health System	Gainesville	Research Assistant	
David	Leehey		MD	Edward Hines Jr. Veterans Administration Hospital	Hines	Director	Network of Dedicated Enrollment Sites (NODES)
Conor	McBurney			Edward Hines Jr. Veterans Administration Hospital	Hines	Research Assistant	Network of Dedicated Enrollment Sites (NODES)
Amod	Jain		MD	VA Pacific Islands Health Care System	Honolulu	Sub-Investigator	
Thomas	Park		MD	VA Pacific Islands Health Care System	Honolulu	Sub-Investigator	

Supplemental Online Content: Nonauthor Collaborators
*First name, last name, and suffix (if applicable) are required and will appear in PubMed.

***First Name and Middle Initial(s)**	***Last Name**	***Suffix (eg, Jr, III)**	Academic Degrees	Institution	Location (city, state/province, country)	Role or Contribution, eg, chair, principal investigator	Group (if more than 1 Group listed in the byline) and/or Subgroup (eg, Steering Committee)
Latonia	Broadwater			VA Pacific Islands Health Care System	Honolulu	Local Study Coordinator	
Hashem	El-Serag		Md, MPH	Michael E. DeBakey VA Medical Center	Houston	Local Site Investigator Mentor	
Yassir	Shaib		MD	Michael E. DeBakey VA Medical Center	Houston	Sub-Investigator	
Eric	Taylor		RN	Michael E. DeBakey VA Medical Center	Houston	Sub-Investigator	
Paayal	Nair		BS, MPH	Michael E. DeBakey VA Medical Center	Houston	Local Study Coordinator	
Emily	Broussard		M.Ed.	Michael E. DeBakey VA Medical Center	Houston	Local Study Coordinator	
Erica	Gibson			Michael E. DeBakey VA Medical Center	Houston	Research Assistant	
Rollin	Hawkins		MS	Michael E. DeBakey VA Medical Center	Houston	Research Assistant	
Tammy	Natividad		MA	Michael E. DeBakey VA Medical Center	Houston	Research Assistant	
Daisy	Courtade			Michael E. DeBakey VA Medical Center	Houston	Research Assistant	
Mayuri (Ezzie)	Palmer			Michael E. DeBakey VA Medical Center	Houston	Research Assistant	
Stephanie	Martinez			Michael E. DeBakey VA Medical Center	Houston	Research Assistant	
Kougias	Panagiotis		MD, MSc	Michael E. DeBakey VA Medical Center	Houston	Director	Network of Dedicated Enrollment Sites (NODES)
Curlie	Morrow		BS	Richard L. Roudebush VA Medical Center	Indianpolis	Local Study Coordinator	

*First name, last name, and suffix (if applicable) are required and will appear in PubMed.

***First Name and Middle Initial(s)**	***Last Name**	***Suffix (eg, Jr, III)**	Academic Degrees	Institution	Location (city, state/province, country)	Role or Contribution, eg, chair, principal investigator	Group (if more than 1 Group listed in the byline) and/or Subgroup (eg, Steering Committee)
Carrie	Ballard		BA	Richard L. Roudebush VA Medical Center	Indianpolis	Local Study Coordinator	
Barry	Barker			Richard L. Roudebush VA Medical Center	Indianapolis	Research Assistant	
Mikayla	Garner			Richard L. Roudebush VA Medica	Indianapolis	Research Assistant	
Stephanie	McCalley		AS	Richard L. Roudebush VA Medical Center	Indianapolis	Research Assistant	
Ashley	Schwartzkopf			Richard L. Roudebush VA Medical Center	Indianapolis	Research Assistant	
Anita	Wright			Richard L. Roudebush VA Medical Center	Indianapolis	Research Assistant	
Mohammad	Titi		MD	Kansas City VA Medical Center	Kansas City	Sub-Investigator	
April	Higbee		RN, BSN	Kansas City VA Medical Center	Kansas City	Local Study Coordinator	
Singh	Pratiksha			Kansas City VA Medical Center	Kansas City	Local Study Coordinator	
April	Jones			Kansas City VA Medical Center	Kansas City	Local Study Coordinator	
Tracey	Shipe			Kansas City VA Medical Center	Kansas City	Research Assistant	
Jason	McKee			Kansas City VA Medical Center	Kansas City	Research Assistant	
Raghuram	Vennalaganti			Kansas City VA Medical Center	Kansas City	Research Assistant	
Kira	Terrey			Kansas City VA Medical Center	Kansas City	Research Assistant	
Lubna	Maruf		MD	Central Arkansas Veterans Healthcare System	Little Rock	Sub-Investigator	

*First name, last name, and suffix (if applicable) are required and will appear in PubMed.

***First Name and Middle Initial(s)**	***Last Name**	***Suffix (eg, Jr, III)**	Academic Degrees	Institution	Location (city, state/province, country)	Role or Contribution, eg, chair, principal investigator	Group (if more than 1 Group listed in the byline) and/or Subgroup (eg, Steering Committee)
Daniel	Brown		MD	Central Arkansas Veterans Healthcare System	Little Rock	Sub-Investigator	
Elaine	Crouse		RN	Central Arkansas Veterans Healthcare System	Little Rock	Sub-Investigator	
Kathy	Marchant-Miros		RN, BSN	Central Arkansas Veterans Healthcare System	Little Rock	Local Study Coordinator	
Betty	Ussery			Central Arkansas Veterans Healthcare System	Little Rock	Local Study Coordinator	
Loretta	Ducker			Central Arkansas Veterans Healthcare System	Little Rock	Local Study Coordinator	
Jennifer	Estell			Central Arkansas Veterans Healthcare System	Little Rock	Local Study Coordinator	
Sylvia	Porchia			Central Arkansa Veterans HS	Little Rock	Research Assistant	
Mi Ye	Kim		DO	VA Loma Linda Healthcare System	Loma Linda	Sub-Investigator	
Christina	Kim		MD	VA Loma Linda Healthcare System	Loma Linda	Sub-Investigator	
Bobby	Chan		MD	VA Loma Linda Healthcare System	Loma Linda	Sub-Investigator	
Amy	Hayton		MD, MPH	VA Loma Linda Healthcare System	Loma Linda	Sub-Investigator	
Harini	Christiansen		Backup NSC	VA Loma Linda Healthcare System	Loma Linda	Local Study Coordinator	
Myra	Peterson		RN	VA Loma Linda Healthcare System	Loma Linda	Local Study Coordinator	
Guizhi (Grace)	Ding			VA Loma Linda Healthcare System	Loma Linda	Local Study Coordinator	
Vicki	Simpson		RN	VA Loma Linda Healthcare System	Loma Linda	Local Study Coordinator	

*First name, last name, and suffix (if applicable) are required and will appear in PubMed.

***First Name and Middle Initial(s)**	***Last Name**	***Suffix (eg, Jr, III)**	Academic Degrees	Institution	Location (city, state/province, country)	Role or Contribution, eg, chair, principal investigator	Group (if more than 1 Group listed in the byline) and/or Subgroup (eg, Steering Committee)
Sonia	Read		LVN	VA Loma Linda Healthcare System	Loma Linda	Local Study Coordinator	
Gabriela	Mamani			VA Loma Linda Healthcare System	Loma Linda	Research Assistant	
Jordan	Salazar			VA Loma Linda Healthcare System	Loma Linda	Research Assistant	
Joann	Kato			VA Loma Linda Healthcare System	Loma Linda	Research Assistant	
Michael	Bowie			VA Loma Linda Healthcare System	Loma Linda	Research Assistant	
Ryan	Carnevale			VA Loma Linda Healthcare System	Loma Linda	Research Assistant	
Mark	Halim			VA Loma Linda Healthcare System	Loma Linda	Research Assistant	
Anita	Sahagian			VA Loma Linda Healthcare System	Loma Linda	Research Assistant	
Allison	Ibarra			VA Loma Linda Healthcare System	Loma Linda	Research Assistant	
Zachary	Travis			VA Loma Linda Healthcare System	Loma Linda	Research Assistant	
Julia	Garcia			VA Loma Linda Healthcare System	Loma Linda	Research Assistant	
Abneet	Gill			VA Loma Linda Healthcare System	Loma Linda	Research Assistant	
John	Starzyk		RN	VA Loma Linda Healthcare Systen	Loma Linda	Research Assistant	
Samantha	Sumarli		RN	VA Loma Linda Healthcare Systen	Loma Linda	Research Assistant	
Valanteena	Johnson			VA Loma Linda Healthcare Systen	Loma Linda	Research Assistant	
Simbiso	Peresuh			VA Loma Linda Healthcare System	Loma Linda	Research Assistant	

*First name, last name, and suffix (if applicable) are required and will appear in PubMed.

***First Name and Middle Initial(s)**	***Last Name**	***Suffix (eg, Jr, III)**	Academic Degrees	Institution	Location (city, state/province, country)	Role or Contribution, eg, chair, principal investigator	Group (if more than 1 Group listed in the byline) and/or Subgroup (eg, Steering Committee)
Traci	Quimby			VA Loma Linda Healthcare System	Loma Linda	Research Assistant	
Rick	Cordova			VA Loma Linda Healthcare System	Loma Linda	Research Assistant	
Lauren	Herold			VA Loma Linda Healthcare System	Loma Linda	Research Assistant	
Angela	Hawley			VA Loma Linda Healthcare System	Loma Linda	Regulatory Manger	
Tyralee	Goo		MD	VA Long Beach Healthcare System	Long Beach	Sub-Investigator	
Jayashri	Kidao		MD	VA Long Beach Healthcare System	Long Beach	Sub-Investigator	
Robert	Lee		MD	VA Long Beach Healthcare System	Long Beach	Sub-Investigator	
Collin	Freidel		BS	VA Long Beach Healthcare System	Long Beach	Local Study Coordinator	
Evelyn	Gutierrez		BS	VA Long Beach Healthcare System	Long Beach	Local Study Coordinator	
Negar	Noorisharghi		BS	VA Long Beach Healthcare System	Long Beach	Local Study Coordinator	
Gabrielle Evanne-Mignon	Ivie		BS/BA	VA Long Beach Healthcare System	Long Beach	Local Study Coordinator	
Tamara	Jawish			VA Long Beach Healthcare System	Long Beach	Local Study Coordinator	
Steven	Partin			VA Long Beach Healthcare System	Long Beach	Local Study Coordinator	
Grace	Yoo			VA Long Beach Healthcare System	Long Beach	Local Study Coordinator	
Charina	Fabilane		BS	VA Long Beach Healthcare System	Long Beach	Local Study Coordinator	

*First name, last name, and suffix (if applicable) are required and will appear in PubMed.

***First Name and Middle Initial(s)**	***Last Name**	***Suffix (eg, Jr, III)**	Academic Degrees	Institution	Location (city, state/province, country)	Role or Contribution, eg, chair, principal investigator	Group (if more than 1 Group listed in the byline) and/or Subgroup (eg, Steering Committee)
Nikoo	Noori		BS	VA Long Beach Healthcare System	Long Beach	Local Study Coordinator	
Elizabeth	Lang		BS	VA Long Beach Healthcare System	Long Beach	Local Study Coordinator	
Shazie	Senen		BS	VA Long Beach Healthcare System	Long Beach	Local Study Coordinator	
Stephen	Partin			VA Long Beach Healthcare System	Long Beach	Research Assistant	
Moe	Fathi		MA	VA Long Beach Healthcare System	Long Beach	Research Assistant	
Rachel	Chauvin			VA Long Beach Healthcare System	Long Beach	Research Assistant	
Jeremy	Feiger			VA Long Beach Healthcare System	Long Beach	Research Assistant	
Marielle	Reataza		MD	VA Long Beach Healthcare System	Long Beach	Research Assistant	
Timothy	Morgan		MD	VA Long Beach Healthcare Sysytem	Long Beach	Director	Network of Dedicated Enrollment Sites (NODES)
Aliya	Asghar			VA Long Beach Healthcare Sysytem	Long Beach	Manager	Network of Dedicated Enrollment Sites (NODES)
Jonathan	Jacobs		MD, PhD	VA Greater Los Angeles Healthcare System (GLA)	Los Angeles	Sub-Investigator	
Mehran	Kashefi		DO	VA Greater Los Angeles Healthcare System (GLA)	Los Angeles	Sub-Investigator	
Folasade	May		MD	VA Greater Los Angeles Healthcare System (GLA)	Los Angeles	Sub-Investigator	
Gordon	Ohning		MD	VA Greater Los Angeles Healthcare System (GLA)	Los Angeles	Sub-Investigator	
David	Padua		MD, PhD	VA Greater Los Angeles Healthcare System (GLA)	Los Angeles	Sub-Investigator	

*First name, last name, and suffix (if applicable) are required and will appear in PubMed.

***First Name and Middle Initial(s)**	***Last Name**	***Suffix (eg, Jr, III)**	Academic Degrees	Institution	Location (city, state/province, country)	Role or Contribution, eg, chair, principal investigator	Group (if more than 1 Group listed in the byline) and/or Subgroup (eg, Steering Committee)
Victoria	Smutko			VA Greater Los Angeles Healthcare System (GLA)	Los Angeles	Local Study Coordinator	
David	Oh		MD	VA Greater Los Angeles Healthcare System (GLA)	Los Angeles	Local Study Coordinator	
Hovsep	Kosoyan		Ph.D.	VA Greater Los Angeles Healthcare System (GLA)	Los Angeles	Local Study Coordinator	
Benjamin	Dreskin			VA Greater Los Angeles Healthcare System (GLA)	Los Angeles	Local Study Coordinator	
Kayti	Luu			VA Greater Los Angeles Healthcare System (GLA)	Los Angeles	Local Study Coordinator	
Alexander	Alas			VA Greater Los Angeles Healthcare System (GLA)	Los Angeles	Local Study Coordinator	
Purnima	Bharath		MA	VA Greater Los Angeles Healthcare System (GLA)	Los Angeles	Local Study Coordinator	
Vivek	Dixit			VA Greater Los Angeles Healthcare System (GLA)	Los Angeles	Local Study Coordinator	
Daniel	Sanford**			VA Greater Los Angeles Healthcare System (GLA)	Los Angeles	Local Study Coordinator	
John	Vu			VA Greater Los Angeles Healthcare System (GLA)	Los Angeles	Local Study Coordinator	
Purnima	Bharath		MA	VA Greater Los Angeles Healthcare System (GLA)	Los Angeles	Research Assistant	
Samar	Kadi		RTT	VA Greater Los Angeles Healthcare System (GLA)	Los Angeles	Research Assistant	
Tracy	Nwajuaku			VA Greater Los Angeles Healthcare System (GLA)	Los Angeles	Research Assistant	
June	Smith		LVN	VA Greater Los Angeles Healthcare System (GLA)	Los Angeles	Research Assistant	
Jenevieve	Kincaid		Dr.	Robley Rex VA Medical Center	Louisville	Local Study Coordinator	

Supplemental Online Content: Nonauthor Collaborators
*First name, last name, and suffix (if applicable) are required and will appear in PubMed.

*First Name and Middle Initial(s)	*Last Name	*Suffix (eg, Jr, III)	Academic Degrees	Institution	Location (city, state/province, country)	Role or Contribution, eg, chair, principal investigator	Group (if more than 1 Group listed in the byline) and/or Subgroup (eg, Steering Committee)
Mohammed	Mir		BDS	Robley Rex VA Medical Center	Louisville	Local Study Coordinator	
Joel	Lanceta			Robley Rex VA Medical Center	Louisville	Research Assistant	
John	Rice		MD	William S. Middleton Memorial Veterans Hospital	Madison	Sub-Investigator	
Parul	Agarwal		MD	William S. Middleton Memorial Veterans Hospital	Madison	Sub-Investigator	
Alexia	Augustine		BA	William S. Middleton Memorial Veterans Hospital	Madison	Local Study Coordinator	
Helene	Prickel			William S. Middleton Memorial Veterans Hospital	Madison	Local Study Coordinator	
Jenny	Vue		BS	William S. Middleton Memorial Veterans Hospital	Madison	Research Assistant	
Kevin	Guerrero			William S. Middleton Memorial Veterans Hospital	Madison	Research Assistant	
Stewart	Levenson		MD	Manchester VA Medical Center	Manchester	Sub-Investigator	
Andres	Aguilera-Fish		MPH	Manchester VA Medical Center	Manchester	Local Study Coordinator	
Leigh	Chestnut			Manchester VA Medical Center	Manchester	Local Study Coordinator	
Bruce	Jobse			Manchester VA Medical Center	Manchester	Local Study Coordinator	
Cathy	Lombardo			Manchester VA Medical Center	Manchester	Local Study Coordinator	
Trudi	Silver			Manchester VA Medical Center	Manchester	Local Study Coordinator	
Nazneen	Ahmed		MD	Memphis VA Medical Center	Memphis	Sub-Investigator	

Supplemental Online Content: Nonauthor Collaborators
*First name, last name, and suffix (if applicable) are required and will appear in PubMed.

*First Name and Middle Initial(s)	*Last Name	*Suffix (eg, Jr, III)	Academic Degrees	Institution	Location (city, state/province, country)	Role or Contribution, eg, chair, principal investigator	Group (if more than 1 Group listed in the byline) and/or Subgroup (eg, Steering Committee)
Kathleen	Bockhold			Memphis VA Medical Center	Memphis	Local Study Coordinator	
Rachel	Koppelman		MD	Miami VA Healthcare System	Miami	Sub-Investigator	
Flavia Rosa	Carneiro		PhD	Miami VA Healthcare System	Miami	Local Study Coordinator	
Rolando	Garcia-Morales			Miami VA Healthcare System	Miami	Local Study Coordinator	
Daniel	Spector			Miami VA Healthcare System	Miami	Local Study Coordinator	
Jose	Gomez			Miami VA Healthcare System	Miami	Research Assistant	
Kyle	Lehenbauer		MD	Minneapolis VA Health Care System	Minneapolis	Sub-Investigator	
Andrew	Reinink		MD	Minneapolis VA Health Care System	Minneapolis	Sub-Investigator	
Ruth	Anway		RN, BA	Minneapolis VA Health Care System	Minneapolis	Local Study Coordinator	
Mary	Evans-Lindquist			Minneapolis VA Health Care System	Minneapolis	Local Study Coordinator	
Thomasine	Eggers		RN	Minneapolis VA Health Care System	Minneapolis	Local Study Coordinator	
Michele	Green		RN	Minneapolis VA Health Care System	Minneapolis	Local Study Coordinator	
Alex	Pretti			Minneapolis VA Health Care System	Minneapolis	Local Study Coordinator	
Tacy	Meyeraan		RN	Minneapolis VA Health Care System	Minneapolis	Local Study Coordinator	
Debra	Condon			Minneapolis VA Health Care System	Minneapolis	Manager	Network of Dedicated Enrollment Sites (NODES)

*First name, last name, and suffix (if applicable) are required and will appear in PubMed.

*First Name and Middle Initial(s)	*Last Name	*Suffix (eg, Jr, III)	Academic Degrees	Institution	Location (city, state/province, country)	Role or Contribution, eg, chair, principal investigator	Group (if more than 1 Group listed in the byline) and/or Subgroup (eg, Steering Committee)
Mary	Cavanagh		MD, MPH	Northport VA Medical Center	Northport	XX	
Colleen M.	DeFalco		MSN, NP	Northport VA Medical Center	Northport	Sub-Investigator	
Jacqueline	Cutting		BSN, RN	Northport VA Medical Center	Northport	Local Study Coordinator	
Kurt	Joseph			Northport VA Medical Center	Northport	Local Study Coordinator	
Stephanie	Dean		MHR	Oklahoma City VA Medical Center	Oklahoma City	Local Study Coordinator	
Stephanie	Mitchell		RN	Oklahoma City VA Medical Center	Oklahoma City	Local Study Coordinator	
Vickie	Phillips			Oklahoma City VA Medical Center	Oklahoma City	Local Study Coordinator	
Chelsey	Krueger			Oklahoma City VA Medical Center	Oklahoma City	Research Assistant	
Donna	Zink		MS	Oklahoma City VA Medical Center	Oklahoma City	Research Assistant	
Kenneth	Goldberg		MD	Orlando VA Medical Center	Orlando	Local Site Investigator Mentor	
Nitin	Patel			Orlando VA Medical Center	Orlando	Local Study Coordinator	
Maria	Martinez Gonzalez			Orlando VA Medical Center	Orlando	Local Study Coordinator	
Heather	Davis-Underwood			Orlando VA Medical Center	Orlando	Local Study Coordinator	
Andrea	O'Sullivan		BA	Orlando VA Medical Center	Orlando	Local Study Coordinator	

*First name, last name, and suffix (if applicable) are required and will appear in PubMed.

***First Name and Middle Initial(s)**	***Last Name**	***Suffix (eg, Jr, III)**	Academic Degrees	Institution	Location (city, state/province, country)	Role or Contribution, eg, chair, principal investigator	Group (if more than 1 Group listed in the byline) and/or Subgroup (eg, Steering Committee)
Trisha	Suppes		MD, PhD	VA Palo Alto Health Care System	Palo Alto	Director	Network of Dedicated Enrollment Sites (NODES)
Karen	Bratcher			VA Palo Alto Health Care System	Palo Alto	Manager	Network of Dedicated Enrollment Sites (NODES)
David	Stern		MD, MSc	Philadelphia VA Medical Center	Philadephia	Sub-Investigator	
Richard	Wu		MD	Philadelphia VA Medical Center	Philadephia	Sub-Investigator	
Jibreel	Damisa		MDHS	Philadelphia VA Medical Center	Philadelphia	Local Study Coordinator	
Rahma	Warsi		BA	Philadelphia VA Medical Center	Philadelphia	Local Study Coordinator	
Tam	Nguyen			Philadelphia VA Medical Center	Philadelphia	Research Assistant	
Hugo	Pinillos		MD	Carl T Hayden VA Medical Center, Phoenix	Phoenix	Sub-Investigator	
Erin	Flowers			Carl T Hayden VA Medical Center	Phoenix	Local Study Coordinator	
Angela	Kuramoto		RT, MHA, CCRC, SCRC	Carl T Hayden VA Medical Center	Phoenix	Local Study Coordinator	
Charles	Thurmond			Carl T Hayden VA Medical Center	Phoenix	Local Study Coordinator	
Lisa	Orozco			Carl T Hayden VA Medical Center	Phoenix	Local Study Coordinator	

Supplemental Online Content: Nonauthor Collaborators
*First name, last name, and suffix (if applicable) are required and will appear in PubMed.

***First Name and Middle Initial(s)**	***Last Name**	***Suffix (eg, Jr, III)**	Academic Degrees	Institution	Location (city, state/province, country)	Role or Contribution, eg, chair, principal investigator	Group (if more than 1 Group listed in the byline) and/or Subgroup (eg, Steering Committee)
Gail	Farrell		CCRC	Carl T Hayden VA Medical Center	Phoenix	Local Study Coordinator	
Rodney	Jaynes		CRC	Carl T Hayden VA Medical Center	Phoenix	Local Study Coordinator	
Perfecto	Saenz			Carl T Hayden VA Medical Center	Phoenix	Research Assistant	
Cassandra	Perez			Carl T Hayden VA Medical Center	Phoenix	Local Study Coordinator	
Lisa	Orozco			Carl T Hayden VA Medical Center	Phoenix	Research Assistant	
Judith	Collins		MD	Portland VA Medical Center	Portland	Sub-Investigator	
Emery	Lin		MD	Portland VA Medical Center	Portland	Sub-Investigator	
Laura	Onstad		RN	Portland VA Medical Center	Portland	Local Study Coordinator	
Alexandra	Pitts			Portland VA Medical Center	Portland	Local Study Coordinator	
Kevin	Osborn			Portland VA Medical Center	Portland	Local Study Coordinator	
Tawni	Kenworthy-Heinige			Portland VA Medical Center	Portland	Research Assistant	
Andrew	Casey			Portland VA Medical Center	Portland	Research Assistant	
Deanna	Gold		MA	Portland VA Medical Center	Portland	Research Assistant	
Cosette	Olivo			Portland VA Medical Center	Portland	Research Assistant	
Alexandra	Pitts			Portland VA Medical Center	Portland	Research Assistant	
Kevin	Osborn			Portland VA Medical Center	Portland	Research Assistant	

Supplemental Online Content: Nonauthor Collaborators
*First name, last name, and suffix (if applicable) are required and will appear in PubMed.

*First Name and Middle Initial(s)	*Last Name	*Suffix (eg, Jr, III)	Academic Degrees	Institution	Location (city, state/province, country)	Role or Contribution, eg, chair, principal investigator	Group (if more than 1 Group listed in the byline) and/or Subgroup (eg, Steering Committee)
Sandra	Joos			Portland VA Medical Center	Portland	Research Assistant	
Sandhya	Subramanian			Portland VA Medical Center	Portland	Research Assistant	
Vanessa	Forro			Portland VA Medical Center	Portland	Research Assistant	
Allina	Cannady			Portland VA Medical Center	Portland	Research Assistant	
Joy	Usih			Portland VA Medical Center	Portland	Research Assistant	
Merritt	Raitt		MD	Portland VA Medical Center	Portland	Director	Network of Dedicated Enrollment Sites (NODES)
Maura	Flynn		MSN, NP	Providence VA Medical Center	Providence	Sub-Investigator	
Yetunde	Shittu		RNP	Providence VA Medical Center	Providence	Sub-Investigator	
Muhammad	Jawaid		MD	Providence VA Medical Center	Providence	Local Study Coordinator	
Rebecca	Anderson			Providence VA Medical Center	Providence	Local Study Coordinator	
Deanna	Poole			Providence VA Medical Center	Providence	Local Study Coordinator	
Celia	Butler			Providence VA Medical Center	Providence	Local Study Coordinator	
Leanne	Snead			Providence VA Medical Center	Providence	Local Study Coordinator	
Hayden	Christine			Providence VA Medical Center	Providence	Research Assistant	
Gina	Swanson			Providence VA Medical Center	Providence	Research Assistant	

Supplemental Online Content: Nonauthor Collaborators
*First name, last name, and suffix (if applicable) are required and will appear in PubMed.

***First Name and Middle Initial(s)**	***Last Name**	***Suffix (eg, Jr, III)**	Academic Degrees	Institution	Location (city, state/province, country)	Role or Contribution, eg, chair, principal investigator	Group (if more than 1 Group listed in the byline) and/or Subgroup (eg, Steering Committee)
Douglas	Heumann		MD	Hunter Holmes McGuire VA Medical Center	Richmond	Sub-Investigator	
William	Pandak		Md	Hunter Holmes McGuire VA Medical Center	Richmond	Sub-Investigator	
Joseph	Spataro		MD	Hunter Holmes McGuire VA Medical Center	Richmond	Sub-Investigator	
Jasmohan	Bajaj		MD	Hunter Holmes McGuire VA Medical Center	Richmond	Sub-Investigator	
Cynthia	Solomon		ACNP	Hunter Holmes McGuire VA Medical Center	Richmond	Sub-Investigator	
HoChong	Smith Gilles		RN, MS, FNP-C	Hunter Holmes McGuire VA Medical Center	Richmond	Sub-Investigator	
Alvin	Zafass		MD	Hunter Holmes McGuire VA Medical Center	Richmond	Sub-Investigator	
Shan	Tilak		MD	hunter Holmes McGuire VA Medical Center	Richmond	Sub-Investigator	
Michael	Fuchs		MD, PhD	Hunter Holmes McGuire VA Medical Center	Richmond	Sub-Investigator	
Margaret	Davis		RN	Hunter Holmes McGuire VA Medical Center	Richmond	Local Study Coordinator	
Lou	Blackman		RN	Hunter Holmes McGuire VA Medical Center	Richmond	Local Study Coordinator	
Jennifer	Lemler		RN	Hunter Holmes McGuire VA Medical Center	Richmond	Local Study Coordinator	
Rachel	Munsey		RN	Hunter Holmes McGuire VA Medical Center	Richmond	Local Study Coordinator	
Jeanette	Deebo			Hunter Holmes McGuire VA Medical Center	Richmond	Local Study Coordinator	
Jane	McCarthy			Hunter Holmes McGuire VA Medical Center	Richmond	Research Assistant	
Megan	Noel			Hunter Holmes McGuire VA Medical Center	Richmond	Research Assistant	

Supplemental Online Content: Nonauthor Collaborators
*First name, last name, and suffix (if applicable) are required and will appear in PubMed.

***First Name and Middle Initial(s)**	***Last Name**	***Suffix (eg, Jr, III)**	Academic Degrees	Institution	Location (city, state/province, country)	Role or Contribution, eg, chair, principal investigator	Group (if more than 1 Group listed in the byline) and/or Subgroup (eg, Steering Committee)
Robin	Hurley		MD, FANPA	W.G. (Bill) Hefner VA Medical Center	Salisbury	Local Site Investigator Mentor	
Wayner	Zurowski		MD	W.G. (Bill) Hefner VA Medical Center	Salisbury	Sub-Investigator	
Angela	Sharpe		ANP	W.G. (Bill) Hefner VA Medical Center	Salisbury	Sub-Investigator	
Krystina	Wilson			W.G. (Bill) Hefner VA Medical Center	Salisbury	Local Study Coordinator	
Risa	Peets		RN	W.G. (Bill) Hefner VA Medical Center	Salisbury	Local Study Coordinator	
Benjamin	Lorenso			W.G. (Bill) Hefner VA Medical Center	Salisbury	Local Study Coordinator	
Stephanie	Burrison		MSN, RN	W.G. (Bill) Hefner VA Medical Center	Salisbury	Local Study Coordinator	
Dorothea	Johnson		LPN	W.G. (Bill) Hefner VA Medical Center	Salisbury	Local Study Coordinator	
Aaron	Angelovic		BSN	VA Salt Lake City Health Care System	Salt Lake City	Local Study Coordinator	
Nathan	Erickson			VA Salt Lake City Health Care System	Salt Lake City	Local Study Coordinator	
Heather	Hanson			VA Salt Lake City Health Care System	Salt Lake City	Local Study Coordinator	
Travis	Bailey			VA Salt Lake City Health Care System	Salt Lake City	Research Assistant	
Kim	Genung		LPN	VA Salt Lake City Health Care System	Salt Lake City	Research Assistant	
Heather	Dulin			VA Salt Lake City Health Care System	Salt Lake City	Research Assistant	

*First name, last name, and suffix (if applicable) are required and will appear in PubMed.

***First Name and Middle Initial(s)**	***Last Name**	***Suffix (eg, Jr, III)**	Academic Degrees	Institution	Location (city, state/province, country)	Role or Contribution, eg, chair, principal investigator	Group (if more than 1 Group listed in the byline) and/or Subgroup (eg, Steering Committee)
Nathan	Erickson			VA Salt Lake City Health Care System	Salt Lake City	Research Assistant	
Renee	Neuharth			VA Salt Lake City Health Care System	Salt Lake City	Research Assistant	
Daniel	Clegg		MD	Salt Lake City VAMC	Salt Lake City	Director	Network of Dedicated Enrollment Sites (NODES)
Kandi	Velarde			Salt Lake City VAMC	Salt Lake City	Manager	Network of Dedicated Enrollment Sites (NODES)
John	Dever			VA San Diego Healthcare System	San Diego	Sub-Investigator	
Mary	Murphy		RN	VA San Diego Healthcare System	San Diego	Local Study Coordinator	
Danielle	Beck		MPH	VA San Diego Healthcare System	San Diego	Local Study Coordinator	
Michelene	Wasil		MA	VA San Diego Healthcare System	San Diego	Local Study Coordinator	
Jason	Vazquez			VA San Diego Healthcare System	San Diego	Local Study Coordinator	
Ellen	O'Neil			VA San Diego Healthcare System	San Diego	Research Assistant	
Gelareh	Ganji			VA San Diego Healthcare System	San Diego	Research Assistant	
Julie	Ducom			VA San Diego Healthcare System	San Diego	Research Assistant	
Maria	Lee			VA San Diego Healthcare System	San Diego	Research Assistant	
Rita	Hovespians			VA San Diego Healthcare System	San Diego	Research Assistant	
Jessie	Margolis			VA San Diego Healthcare System	San Diego	Research Assistant	

Supplemental Online Content: Nonauthor Collaborators
*First name, last name, and suffix (if applicable) are required and will appear in PubMed.

***First Name and Middle Initial(s)**	***Last Name**	***Suffix (eg, Jr, III)**	Academic Degrees	Institution	Location (city, state/province, country)	Role or Contribution, eg, chair, principal investigator	Group (if more than 1 Group listed in the byline) and/or Subgroup (eg, Steering Committee)
Brittni	Simmons		BA	VA San Diego Healthcare System	San Diego	Research Assistant	
Jennifer	Sweeney (formerly St. Martin)			VA San Diego Healthcare System	San Diego	Research Assistant	
Robert	Henry		MD	VA San Diego Healthcare System	San Diego	Director	Network of Dedicated Enrollment Sites (NODES)
Jose	Martin-Ortiz		MD	VA Caribbean Healthcare System	San Juan	Sub-Investigator	
Loscar	Santiago-Rivera		MD	VA Caribbean Healthcare System	San Juan	Sub-Investigator	
Gladys	Gonzalez - Aviles		MS	VA Caribbean Healthcare System	San Juan	Local Study Coordinator	
Carmen	Pedrosa		BS	VA Caribbean Healthcare System	San Juan	Local Study Coordinator	
Juan	Bird-Caceres		MD	VA Caribbean Healthcare System	San Juan	Research Assistant	
Brunilda	Padilla			VA Caribbean Healthcare System	San Juan	Research Assistant	
Fernando	Baez-Corujo		MD	VA Caribbean Healthcare System	San Juan	Research Assistant	
Yaiza	Martinez- Ortiz		MD	VA Caribbean Healthcare System	San Juan	Research Assistant	
Ekie	Vazquez		MD	VA Caribbean Healthcare System	San Juan	Research Assistant	
Nicole	Grigg-Gutierrez		MD	VA Caribbean Healthcare System	San Juan	Research Assistant	
Carlos	Laboy-Olivieri		MD	VA Caribbean Healthcare System	San Juan	Research Assistant	
Rafael	Urbina-Velez		MD	VA Caribbean Healthcare System	San Juan	Research Assistant	

Supplemental Online Content: Nonauthor Collaborators
*First name, last name, and suffix (if applicable) are required and will appear in PubMed.

***First Name and Middle Initial(s)**	***Last Name**	***Suffix (eg, Jr, III)**	Academic Degrees	Institution	Location (city, state/province, country)	Role or Contribution, eg, chair, principal investigator	Group (if more than 1 Group listed in the byline) and/or Subgroup (eg, Steering Committee)
Sarah	Andrews		MD	VA Caribbean Healthcare System	San Juan	Research Assistant	
Sheryl	Rosa-Cruz		MD	VA Caribbean Healthcare System	San Juan	Research Assistant	
Rafael	Tirado-Montijo		MD	VA Caribbean Healthcare System	San Juan	Research Assistant	
Kayssa	Otero-Aponte		MPH	VA Caribbean Healthcare System	San Juan	Research Assistant	
Michelle	Martinez-Martino		MPH	VA Caribbean Healthcare System	San Juan	Research Assistant	
Arnaldo	Freire		MD	VA Caribbean Healthcare System	San Juan	Research Assistant	
Antonio	Soto-Ramos		MD	VA Caribbean Healthcare System	San Juan	Research Assistant	
Margarita	Santana-Perez		MBA	VA Caribbean Healthcare System	San Juan	Research Assistant	
James	Araujo		MD	VA Puget Sound Health Care Syst	Seattle	Sub-Investigator	
Christopher	Vanderwarker		MD	VA Puget Sound Health Care System	Seattle	Sub-Investigator	
Jeremiah	Alexander		MD	VA Puget Sound Health Care System	Seattle	Sub-Investigator	
Elaine	Nevins			VA Puget Sound Health Care System	Seattle	Local Study Coordinator	
Julie	LaGuire		RN	VA Puget Sound Health Care System	Seattle	Local Study Coordinator	
Linda	Guerrero			VA Puget Sound Health Care System	Seattle	Local Study Coordinator	
Gina	Piehl			VA Puget Sound Health Care System	Seattle	Research Assistant	
Meredith	Jenkins			VA Puget Sound Health Care System	Seattle	Research Assistant	

*First Name and Middle Initial(s)	*Last Name	*Suffix (eg, Jr, III)	Academic Degrees	Institution	Location (city, state/province, country)	Role or Contribution, eg, chair, principal investigator	Group (if more than 1 Group listed in the byline) and/or Subgroup (eg, Steering Committee)
Teresa	Chen			VA Puget Sound Health Care System	Seattle	Research Assistant	
Jessica	Jang			VA Puget Sound Health Care System	Seattle	Research Assistant	
Adrienne	Tanus			VA Puget Sound Health Care System	Seattle	Research Assistant	
Patricia	McKelvy		MD	St. Louis VA Medical Center	St. Louis	Sub-Investigator	
Gregory	Sayuk		MD, MPH	St. Louis VA Medical Center	St. Louis	Sub-Investigator	
Pooja	Chandiramani			St. Louis VA Medical Center	St. Louis	Local Study Coordinator	
Lindsey	Vargo (Formerly Shoults)			St. Louis VA Medical Center	St. Louis	Local Study Coordinator	
Kristin	Vargo			St. Louis VA Medical Center	St. Louis	Local Study Coordinator	
Sara	Lawrence		PA-C	St. Louis VA Medical Center	St. Louis	Research Assistant	
Kelly	Blythe			St. Louis VA Medical Center	St. Louis	Research Assistant	
Susan	Goldsmith		MD	James A. Haley Veterans' Hospital	Tampa	Sub-Investigator	
Robert	Campbell		MD	James A. Haley Veterans' Hospital	Tampa	Sub-Investigator	
Mary Agnes	Craddock		APRN	James A. Haley Veterans' Hospital	Tampa	Sub-Investigator	
Donald	Amodeo		MD	James A. Haley Veterans' Hospital	Tampa	Local Site Investigator Mentor	

Supplemental Online Content: Nonauthor Collaborators
*First name, last name, and suffix (if applicable) are required and will appear in PubMed.

*First Name and Middle Initial(s)	*Last Name	*Suffix (eg, Jr, III)	Academic Degrees	Institution	Location (city, state/province, country)	Role or Contribution, eg, chair, principal investigator	Group (if more than 1 Group listed in the byline) and/or Subgroup (eg, Steering Committee)
Rebecca	Reinhard		RN	James A. Haley Veterans' Hospital	Tampa	Local Study Coordinator	
Brittany	Durant			James A. Haley Veterans' Hospital	Tampa	Local Study Coordinator	
Breanna	Wells		LPN	James A. Haley Veterans Hospital	Tampa	Research Assistant	
Adam	Zoble			James A. Haley Veterans Hospital	Tampa	Research Assistant	
Marianna	Papademetriou		MD	Washington DC VA Medical Center	Washington, DC	Sub-Investigator	
Nada	Roche		MD	Washington DC VA Medical Center	Washington, DC	Sub-Investigator	
Priscilla	Adler			Washington DC VA Medical Center	Washington, DC	Local Study Coordinator	
Asha	Krishna			Washington DC VA Medical Center	Washington, DC	Local Study Coordinator	
Delmonica	Glaze		RN, CCRC	Washington DC VA Medical Center	Washington, DC	Local Study Coordinator	
Dinuka	Wijegunawardana			Washington DC VA Medical Center	Washington, DC	Local Study Coordinator	
Samuel	Yeroushalmi			Washington DC VA Medical Center	Washington, DC	Research Assistant	
Loren	Laine		MD	VA Connecticut Health Care System	West Haven	Sub-Investigator	
Raeleen	Mautner		PhD	VA Connecticut Healthcare System, West Haven Campus	West Haven	Local Study Coordinator	
Julie	Bugaj		MS	VA Connecticut Healthcare System, West Haven Campus	West Haven	Research Assistant	

Supplemental Online Content: Nonauthor Collaborators
*First name, last name, and suffix (if applicable) are required and will appear in PubMed.

***First Name and Middle Initial(s)**	***Last Name**	***Suffix (eg, Jr, III)**	Academic Degrees	Institution	Location (city, state/province, country)	Role or Contribution, eg, chair, principal investigator	Group (if more than 1 Group listed in the byline) and/or Subgroup (eg, Steering Committee)
Joseph	Anderson		MD	White River Junction VA Medical Center	White River Junction	Sub-Investigator	
Andres	Aguilera-Fish		MPH	White River Junction VA Medical Center	White River Junction	Local Study Coordinator	
Bruce	Jobse			White River Junction VA Medical Center	White River Junction	Local Study Coordinator	
Cathy	Lombardo			White River Junction VA Medical Center	White River Junction	Local Study Coordinator	
Trudi	Silver			White River Junction VA Medical Center	White River Junction	Local Study Coordinator	
Heike	Croteau			White River Junction VA Medical Center	White River Junction	Local Study Coordinator	
Linda	Kingman			White River Junction VA Medical Center	White River Junction	Research Assistant	
Laurie	Burridge			White River Junction VA Medical Center	White River Junction	Research Assistant	
