# Supplementary material for: Baseline Features and Reasons for Nonparticipation in the Colonoscopy Versus Fecal Immunochemical Test in Reducing Mortality From Colorectal Cancer (CONFIRM) Study, a Colorectal Cancer Screening Trial
Source: JAMA Netw Open. 2023 Jul 11;6(7):e2321730. doi: 10.1001/jamanetworkopen.2023.21730 (PMC10336619; doi:10.1001/jamanetworkopen.2023.21730)
Supplement: Supplement 3. — Data Sharing Statement [file jamanetwopen-e2321730-s003.pdf]

Data Sharing Statement

Robertson. Baseline Features and Reasons for Nonparticipation in the Colonoscopy Versus Fecal Immunochemical Test in Reducing Mortality From Colorectal Cancer (CONFIRM) Study, a Colorectal Cancer Screening Trial. *JAMA Netw Open*. Published July 11, 2023. doi:10.1001/jamanetworkopen.2023.21730

Data
Data available: No

Additional Information
Explanation for why data not available: The study is ongoing. We are currently reporting the baseline characteristics of the participants and information on those choosing not to participate
